# Systematic Design and Evaluation of Nasal Drug Delivery for Central Nervous System Disease from Experimental to Clinical Application

**DOI:** 10.3390/pharmaceutics18080916

**Published:** 2026-07-25

**Authors:** Xi-Rui Zhou, Yi Zhang, Qianqian Kong, Ziyue Wang, Yiming Luo, Hao Huang, Wensheng Qu, Zhiyuan Yu, Xiang Luo

**Affiliations:** 1 Department of Neurology, Tongji Hospital, Tongji Medical College, Huazhong University of Science and Technology, Wuhan 430030, China; xg1101735313@126.com (X.-R.Z.); haohuang_tj@hust.edu.cn (H.H.); qws0309@163.com (W.Q.); zhiyuan_yu@126.com (Z.Y.); 2 Hubei Key Laboratory of Neural Injury and Functional Reconstruction, Huazhong University of Science and Technology, Wuhan 430030, China; 3 Hubei Provincial Clinical Research Center for Stroke, Wuhan 430030, China

**Keywords:** central nervous system, blood–brain barrier, nasal delivery system, olfactory nerve pathway, trigeminal nerve pathway, molecular biology, drug design, clinical evaluation, clinical application

## Abstract

Disorders of the central nervous system (CNS) are intricate and often resistant conditions that create a significant global impact, affecting millions of individuals each year. The blood–brain barrier (BBB) acts as a protective mechanism for the brain against external substances, but it also prevents most therapeutic agents from entering the CNS, leading to inadequate drug absorption and reduced effectiveness after diagnosis. Nasal drug delivery has emerged as a viable approach to bypass the BBB, facilitating direct access to the brain through the olfactory and trigeminal nerve routes. Although considerable research focuses on innovative nasal formulations with proven clinical promise, a critical gap persists: a systematic framework that bridges laboratory breakthroughs with clinical implementation. This review addresses this unmet need by integrating recent basic research advances with practical clinical requirements. We summarize nasal transport pathways, targeted design strategies, formulation optimization, and device engineering. Crucially, we propose a structured clinical evaluation framework built upon five essential pillars: targeting precision, pharmacokinetic performance, multi-organ safety profiling, device–drug clinical compatibility, and anatomical translation from animal models to humans. By mapping current research capabilities against clinical readiness criteria, this framework identifies translational bottlenecks and provides actionable guidance to accelerate the bench-to-bedside transition of intranasal drug delivery systems for CNS disorders.

## 1. Introduction

According to a report from the World Health Organization (WHO), more than 10 million new cases of central nervous system (CNS) fatalities occur each year globally [1]. Conditions such as Alzheimer’s disease [2,3], epilepsy [4,5], and neural tumors [6,7] exhibit intricate pathogenic processes that make them resistant to effective treatments, imposing significant physical and psychological burdens on patients and their families, as well as substantial economic strain on healthcare systems worldwide [8]. Compounding the issue is the blood–brain barrier (BBB), which serves as the primary interface for the exchange between blood and brain, blocking 99% of small molecules from entering the brain and diminishing the effectiveness of targeted drug delivery [9,10]. This barrier presents a significant challenge to the clinical success of therapies for CNS disorders. To better understand how substances can traverse the BBB, numerous studies have been conducted, investigating various transmembrane pathways and their regulatory mechanisms. These include receptor- or carrier-mediated transport that relies on specific interactions at the BBB surface [11,12], adsorptive-mediated endocytosis driven by electrostatic forces [13,14], and the modulation of tight junction proteins that influence BBB permeability [15,16]. However, CNS pathologies can disrupt drug passage through the BBB and modify its permeability, leading to increased treatment failure rates, which underscores the pressing need for innovative drug delivery methods [17,18].

Nasal administration has emerged as a promising method for delivering drugs effectively to the brain, thereby circumventing the BBB and offering a potential treatment for CNS disorders [19,20]. This approach utilizes the distinctive anatomical features of the nasal cavity, allowing drugs to penetrate the BBB via the olfactory and trigeminal nerve pathways, as well as through systemic absorption via the blood vessels in the nasal mucosa [21,22]. This dual mechanism not only mitigates the first-pass metabolism associated with oral routes and minimizes gastrointestinal discomfort, but also significantly lowers the systemic toxicity of medications, thereby enhancing their bioavailability in the brain [23,24]. The benefits of nasal delivery are clear, including ease of administration, high drug efficacy, and minimal side effects, which have attracted considerable attention in clinical research [25,26]. In recent years, a variety of nasal drug delivery systems have been widely developed, including microspheres [27,28], liposomes [29,30], and hydrogels [31,32]. These systems aim to enhance drug stability within the nasal cavity and extend their retention time, further improving targeted delivery to the brain [33]. While these contributions have been valuable, they remain largely focused on individual technical components and do not provide a systematic pathway to translate laboratory findings into clinical practice [34,35]. A critical unmet need therefore persists: a structured framework that connects recent basic research breakthroughs with bedside clinical requirements is essential to identify translational bottlenecks and accelerate the clinical adoption of intranasal delivery systems [36].

Unlike previous reviews that cataloged individual technologies in isolation, the present work deliberately bridges recent basic research breakthroughs with clinical application needs. This review aims to provide a thorough overview of the design approaches for intranasal drug delivery systems and establishes a systematic clinical evaluation framework to guide their translation into practice. The goal is to pinpoint and summarize the current obstacles, thereby facilitating the clinical application and advancement of these systems. We systematically investigate design and optimization strategies for intranasal medications, encompassing targeted design [37,38], loading design [39,40], and the use of biocompatible materials [41,42], which correspond to enhancing delivery specificity, sustaining effective therapeutic levels and improving clinical safety, respectively. We also discuss the refinement of delivery methods, including the optimization of devices and dosages according to nasal physiological characteristics to improve drug absorption efficiency [43,44]. The central contribution of this review is a structured clinical evaluation framework built upon the following essential pillars: targeting precision, pharmacokinetic performance, multi-organ safety profiling, device–drug clinical compatibility, and anatomical translation from animal models to humans. By mapping current research capabilities against clinical readiness criteria, this framework identifies key translational bottlenecks and provides actionable guidance for accelerating the clinical adoption of intranasal delivery systems. Furthermore, in light of the current advancements in pharmaceuticals [45,46], materials science [47,48], and molecular biology [49,50], we also explore the future prospects of intranasal drug delivery systems, offering insights for the clinical management of central nervous system disorders.

## 2. Central Nervous System Diseases and Brain Barriers

Disorders of the CNS, which include conditions like Alzheimer’s and Parkinson’s diseases [51], neuroinflammatory disorders [52], primary and metastatic brain tumors [53], and cerebrovascular issues [54], represent a major global health challenge, resulting in over 10 million fatalities annually [1]. The complex biological processes involved in these conditions, along with the shortcomings of standard treatment approaches, result in significant obstacles for effective clinical care [55,56]. Surgical procedures can lead to serious postoperative complications and negative side effects; worse still, the postoperative treatment of drug delivery frequently fails to reach targeted sites in CNS disorders [57,58].

### 2.1. Transmembrane Mechanisms of the Blood–Brain Barrier

**Figure 1:**
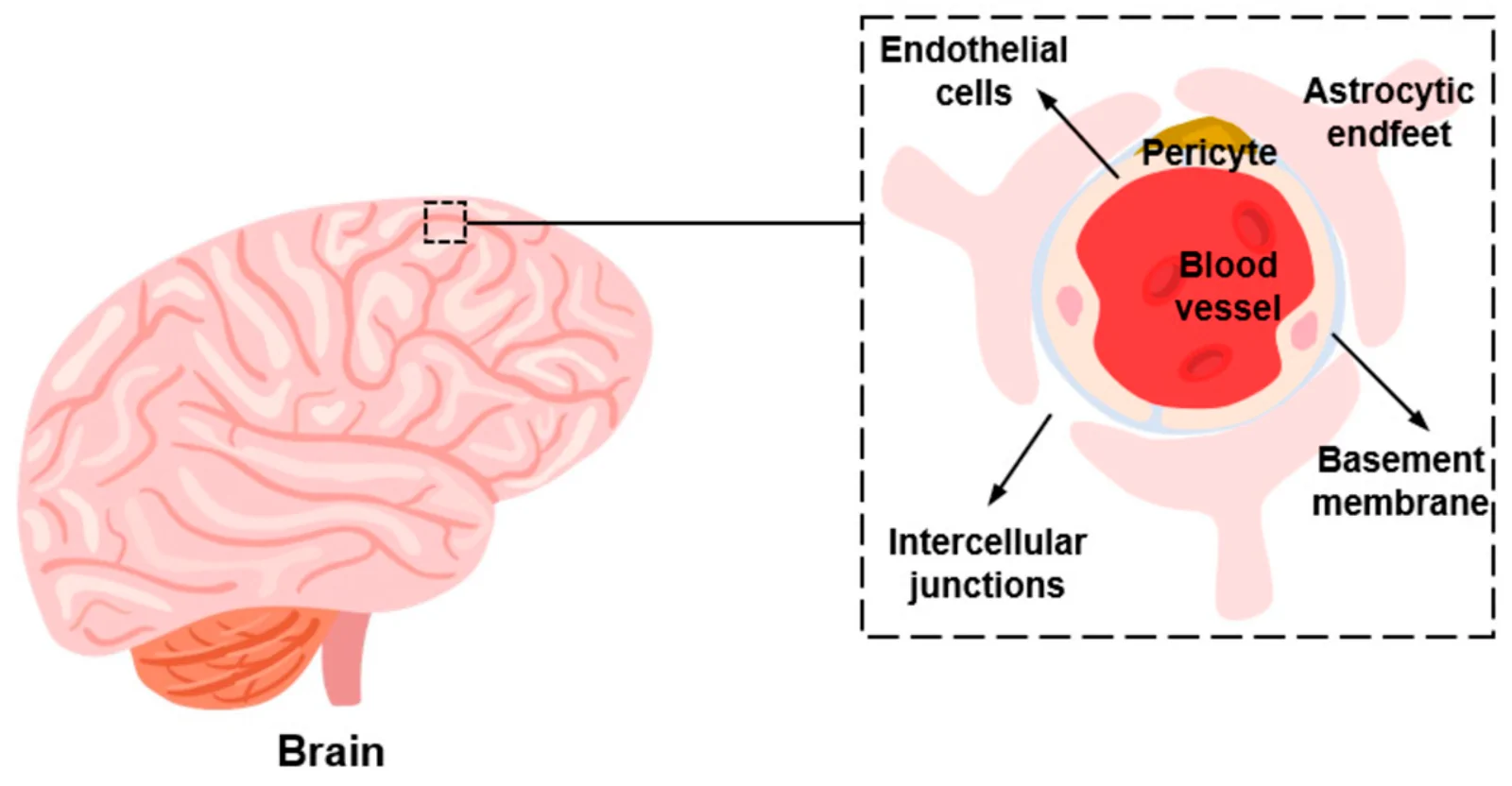
Schematic diagram of the brain and the structure of BBB, which is composed of astrocytes, basement membrane, endothelial cells, and pericytes.

A significant challenge in delivering drugs to the CNS is the presence of the BBB. As illustrated in Figure 1, the BBB consists of a highly specialized neurovascular structure that plays a crucial role in maintaining the stability of the brain’s environment. It is formed by endothelial cells linked through tight junctions, along with astrocytic end-feet, pericytes, and a continuous basement membrane [59]. This distinctive configuration effectively limits the movement of most therapeutic compounds, with estimates suggesting that around 99% of small-molecule medications and nearly all large-molecule biologics cannot penetrate the BBB to achieve effective concentrations in the brain tissue [60].

Table 1 depicts the processes by which molecules traverse the BBB. Most therapeutic compounds possess a high molecular weight, hindering their ability to passively diffuse through biological membranes, a method applicable to smaller molecules such as gases or water [61]. Consequently, the development of efficient BBB-penetrating strategies has emerged as a critical unmet need in neurotherapeutics. In recent years, active transport strategies—encompassing receptor-mediated transcytosis (RMT), exosome-mediated delivery, cell-derived vesicles, transporter engineering, and BBB modulation—have achieved remarkable progress, demonstrating substantial potential for transitioning from passive diffusion to precision-targeted CNS drug delivery. RMT involves specific ligands attaching to receptors on the luminal surface of BBB endothelial cells, initiating endocytosis and subsequent vesicular transport that delivers biomimetic drugs into the brain, such as the transferrin receptor (TfR) and low-density lipoprotein receptor-related protein 1, triggering clathrin-mediated endocytosis and subsequent transcytosis [11]. For example, Benjamin et al. [62] reported a reprogrammed adeno-associated virus capsid that binds human TfR with high specificity, enabling brain-wide gene delivery across the BBB, while Nathalie et al. [63] employed Fc-engineered antibodies simultaneously targeting TfR and CD98hc to significantly enhance the transcytosis efficiency of large-molecule therapeutics. Due to its precise targeting, effective transport capabilities, and favorable safety profile, this method has been extensively researched for BBB penetration [64].

Exosomes (30–150 nm) and cell-derived vesicles leverage their inherent biocompatibility, low immunogenicity, and membrane fusogenicity to traverse the BBB via receptor-mediated uptake and direct transcytosis [65]. Specifically, Jin et al. [66] systematically elaborated the full-spectrum engineering strategies of exosomes as precision tools for CNS-targeted drug delivery, encompassing surface functionalization, cargo loading, and in vivo tracking. Concurrently, Kim et al. [67] demonstrated that ginseng-derived exosome-like nanoparticles actively penetrate the BBB and remodel the glioma microenvironment, offering a plant-based alternative for brain-targeted delivery. Transporter engineering strategies repurpose endogenous BBB machineries—including solute carrier transporters, the neonatal Fc receptor, and major facilitator superfamily domain-containing protein 2A—to augment substrate recognition or construct allosteric delivery platforms [68,69]. For example, Tang et al. [70] proposed an allosteric targeting strategy that mimics transmembrane domain interactions to achieve specific modulation of brain endothelial transporters, and Julien et al. [71] leveraged neonatal Fc receptor engineering to substantially enhance antibody transport across the BBB without compromising barrier integrity. Finally, BBB modulation strategies employ physical (e.g., focused ultrasound), chemical (e.g., *WNT* surrogates), or biological approaches to transiently and reversibly increase BBB permeability, creating a therapeutic window for drug entry [72]. For instance, Ding et al. [73] developed a bioengineered frizzled class receptor 4 selective *WNT* surrogate that activates the *WNT*/β-catenin signaling pathway, achieving therapeutic BBB modulation and marked functional improvement in a stroke model.

Collectively, these strategies each possess distinct mechanistic advantages and clinical trade-offs that necessitate tailored application according to specific neurological pathologies. Building on these findings, researchers have devised various innovative methods to improve BBB penetration, including the engineering of BBB-penetrating peptides [74,75], drug conjugation to transferrin or receptor-specific antibodies [76,77], the creation of prodrugs that utilize carrier transporters [78,79], and the development of nanocarrier systems [80,81]. Preclinical studies have shown promising outcomes, with several of these strategies resulting in significantly enhanced brain exposure, improved therapeutic efficacy, and reduced systemic toxicity in models of central nervous system disorders [82,83].

**Table 1:** Pathways and characteristics of substances crossing the BBB.

Pathways Crossing the BBB	Transported Substance	Advantages	Limitations	Translational Barriers	Clinical Applicability	Ref.
Passively Diffusion	Lipophilic Small Molecules	Simple and Rapid Transport Process	Incapable of Active Transport	Inability to Achieve Active Transport	Clinical Application is Limited	[61]
Receptor-Mediated Transcytosis	Molecules Conjugated with Specific Ligands	High Efficiency, Specificity, Targeting Ability	Prone to Transport Saturation	Complex Ligand Conjugation Chemistry; Receptor Expression Variability Across Patients	CNS Gene Therapy; Targeted Antibody Delivery for Neurodegenerative Diseases	[11]
Exosome-Mediated Delivery	Small-Molecule Drugs; Proteins; miRNA/siRNA/mRNA; Peptides	Natural BBB-Crossing Ability; Low Immunogenicity; Long Circulation	Low Drug Loading; Vesicle Heterogeneity; Purification Challenges	Standardized Isolation Protocols Lacking; Scalability Limited; Storage Stability Concerns	Neurodegenerative Disease Therapy; Glioma-targeted Drug Delivery	[65]
Cell-Derived Vesicles	Cytokines; Growth Factors; Therapeutic Proteins; Genetic Materials; Small Molecules	Inherent Targeting to Injured Brain; Dual Therapeutic/Regenerative Effects	Batch-to-Batch Variability; Undefined Composition; Limited Cargo Loading Control	Autologous Sourcing Complexity; Donor Cell Variability; Batch Consistency Requirements	Stroke Recovery; Traumatic Brain Injury; Neuroinflammation Modulation	[84]
Transporter Engineering	Antibodies; Recombinant Proteins; Peptide Conjugates	Native Transport Exploitation; BBB Integrity Preservation	Complex Engineering; Off-Target Substrate Effects; Allosteric Regulation Poorly Understood	Regulatory Approval for Modified Proteins; Immunogenicity Risk of Engineered Variants	Monoclonal Antibody CNS Delivery; Enzyme Replacement Therapy	[68]
BBB Modulation	Broad Drug Classes: Small Molecules; Antibodies; Nanoparticles; Viral Vectors	Reversible Permeability; Combinable with Other Strategies; Wide Applicability	Neurotoxicity Risk; Narrow Therapeutic Window; Cerebral Edema Potential	Real-Time BBB Permeability Monitoring Needed; Long-Term Safety Data Insufficient	Glioblastoma Chemotherapy Enhancement; Stroke Thrombolysis; Gene Therapy	[72]

### 2.2. Blood–Brain Barrier Dysfunction in CNS Disorders

Disorders of the CNS can cause a breakdown in the integrity of the BBB, leading to increased permeability. This occurs due to the activation of inflammatory processes, oxidative damage, and harm to microvascular structures [85,86]. Consequently, this can result in the leakage of plasma, swelling in the brain, and heightened intracranial pressure, all of which greatly impair the delivery of medications to the brain and pose significant challenges to the effective treatment of central nervous system disorders [87,88].

Alzheimer’s disease affects the tight junctions of the BBB and alters the neurovascular unit’s structure [89], which hinders the function of receptors responsible for clearing amyloid-beta and drug transporters [90]. This condition, in conjunction with reduced cerebral blood flow and neuroinflammation, compromises the integrity of the BBB [91], making it difficult for medications to cross and reach their central targets, significantly lowering the efficiency of drug delivery to the brain [92]. Additionally, seizures can acutely disrupt BBB tight junctions and lead to long-term damage to the neurovascular unit, increasing the activity of drug efflux pumps and causing ongoing neuroinflammation [93]. This results in abnormal drug leakage, rapid removal, and uneven distribution [94], ultimately diminishing the effectiveness of antiepileptic medications on their intended targets [95].

**Figure 2:**
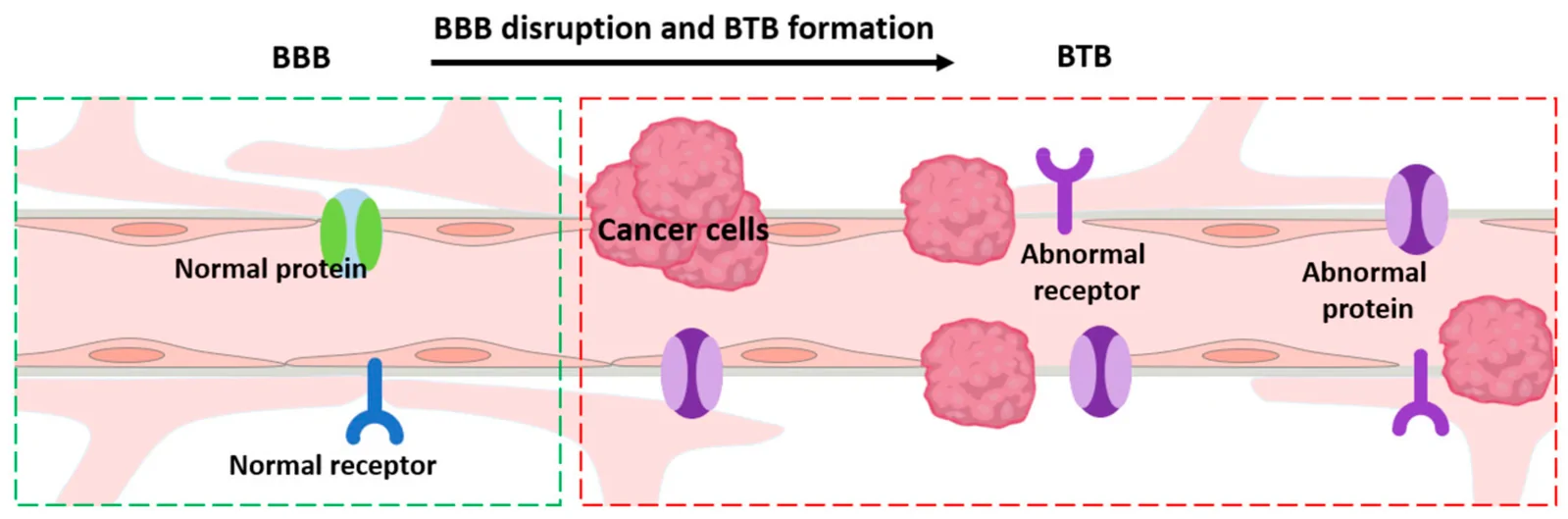
Schematic of BBB structural disruption causing BTB formation and increased vascular permeability induced by neural tumors.

In the case of neural tumors, as illustrated in Figure 2, the disruption of the BBB can occur, which subsequently gives rise to the blood–tumor barrier (BTB). This pathological barrier, resulting from BBB compromise, further weakens the integrity of endothelial cell tight junctions [96,97]. This disruption can lead to the loss or malfunction of pericytes and astrocytes, causing chaotic vascular formations and a notable increase in overall permeability [98]. As a result, the presence of the BTB hinders the effective transmembrane delivery of therapeutic agents, primarily through three interconnected mechanisms [99]. First, the BTB modifies vascular permeability, resulting in highly variable drug distribution and potentially hindering the successful transport of medications to targeted brain lesions [100]. Second, the abnormal accumulation of matrix elements like collagen and glycosaminoglycans within the BTB environment raises the physical barriers to drug diffusion, obstructing the movement of drugs from the blood vessels into adjacent tumor tissues [101]. Lastly, the rapid growth of tumor cells within the BTB elevates internal pressure, creating a pressure differential from the tumor’s interior to its exterior [99]. This pressure gradient directly impedes the effective penetration of drugs from the vascular space into the tumor parenchyma, thereby significantly diminishing the effectiveness of anti-tumor therapies [102,103].

## 3. Intranasal Drug Delivery System

In clinical research on CNS disorders, the selection and development of drug delivery routes are critical. The most frequently used methods include oral, intravenous, and nasal routes [25,35]. Oral and intravenous methods are prevalent for treating CNS conditions due to their established clinical use and ease of administration [58,104]. Nevertheless, as previously mentioned, both methods face challenges from dual biological barriers in CNS treatment, resulting in suboptimal drug absorption [35,99]. Additionally, these delivery routes tend to have inadequate targeting of the CNS, leading to insufficient drug concentrations in the brain [105,106]. The systemic distribution of these drugs via the bloodstream can also lead to unwanted peripheral toxicity and side effects [107,108]. Consequently, they are not the optimal administration routes for the treatment of CNS diseases [59,109].

### 3.1. High Targeting Specificity

The intranasal route is a significant method for delivering drugs aimed at treating CNS conditions [110,111]. This approach merges the benefits of both systemic and localized drug administration, offering accurate targeting [112], quick-action onset [113], efficient drug usage [114], and minimal toxicity [115], making it highly promising for widespread clinical use in CNS therapies [25,116]. Table 2 presents a comprehensive comparison of brain-targeting parameters across CNS-acting drugs formulated for intranasal delivery. As illustrated, intranasal administration conferred substantial improvements in brain exposure and targeting specificity relative to conventional routes. Notably, drug targeting efficiency (DTE) exhibited marked elevations, ranging from 117% (clozapine micelles) to 5451.8% (zolmitriptan bilosomes) [117,118], underscoring the preferential distribution of nasally delivered therapeutics to the brain. Concordantly, the drug targeting index (DTI) values (3.58–144.58) [119,120] and drug transport percentage (DTP) (up to 99.3%) [121] further corroborated the enhanced selectivity of intranasal formulations toward cerebral tissues. The brain-to-plasma maximum observed concentration (C_max_) ratios (0.70–9.85) demonstrated reduced systemic drug exposure [120,122], while relative brain bioavailability values substantially exceeded 100% in multiple formulations (reaching 1173.64% for zolmitriptan) [118], indicating efficient direct nose-to-brain transport that bypasses the systemic circulation. Collectively, these parameters provide compelling evidence that intranasal delivery, particularly when combined with advanced nanocarrier systems, significantly enhances brain targeting efficiency and offers a promising non-invasive strategy for CNS drug delivery.

### 3.2. Intranasal Transport Route

**Figure 3:**
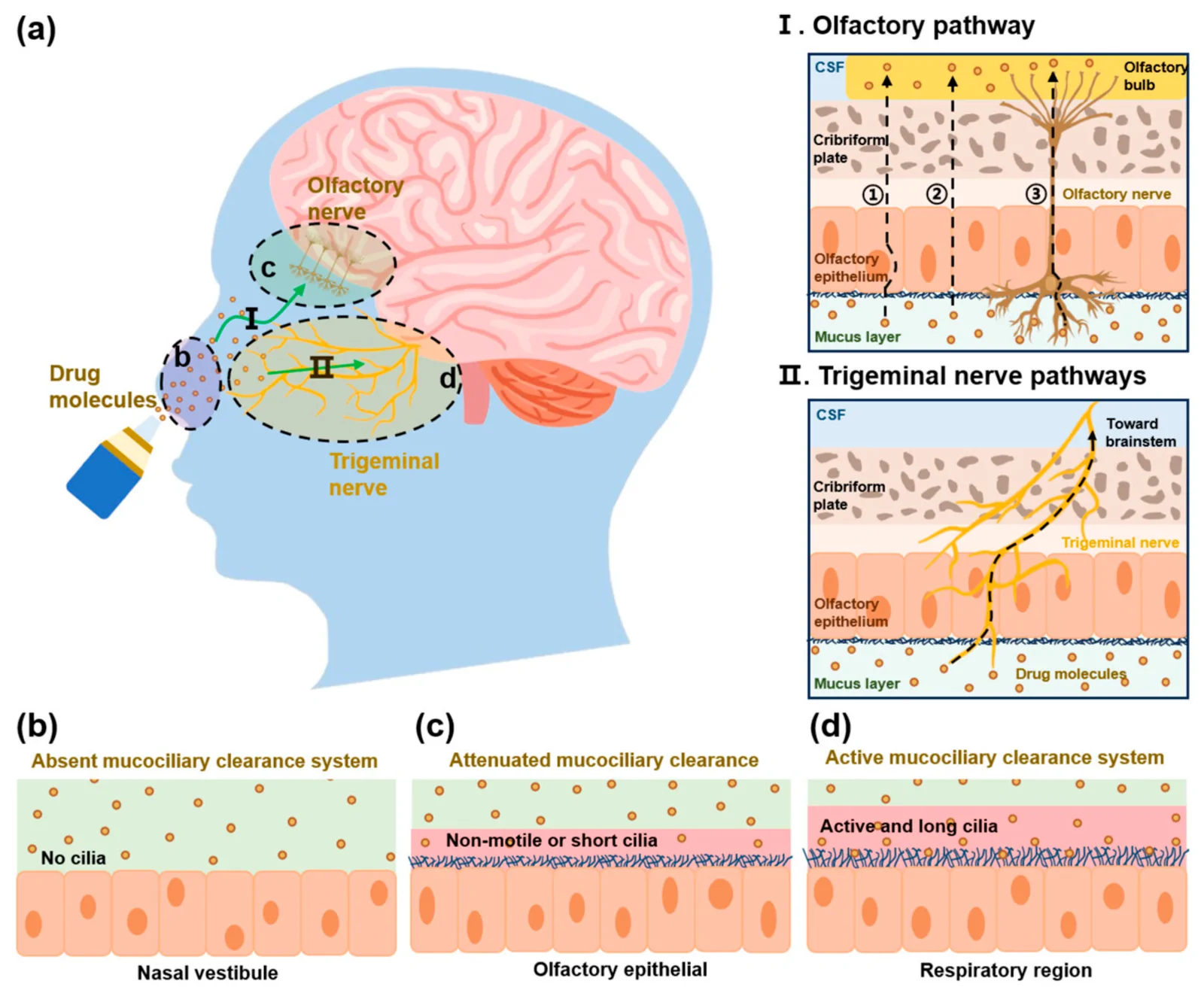
(**a**) Drug molecule delivery routes of the (**I**) olfactory pathway(including ① transcellular pathway, ② paracellular pathway, ③ olfactory nerve axonal pathway) and (**II**) respiratory pathway (trigeminal nerve pathway), and the distinct deposition regions of nasal medications. All arrows indicate drug transport direction. Regional differences in the ciliary clearance activity of drug deposition in the nasal cavity for the (**b**) nasal vestibule, (**c**) olfactory region, and (**d**) respiratory region. Red: mucociliary clearance area; Green: respiratory tract.

Upon intranasal delivery (Figure 3a), the medication moves from the nasal vestibule into the nasal cavity. The nasal vestibule is lined with stratified squamous epithelium and lacks cilia, resulting in an almost absent mucociliary clearance system (Figure 3b). Consequently, drug removal in this area relies mainly on external forces and evaporation, leading to lower clearance efficiency [126,127]. The drug then diffuses into both the olfactory and respiratory regions, where delivery to the brain is primarily facilitated through the olfactory and trigeminal nerve pathways (pathway I and II in Figure 3a), bypassing the BBB to target specific brain areas for localized treatment [128,129]. As shown in Figure 3c, the olfactory epithelial cells in this region have only non-motile or short cilia, contributing to slower drug clearance and an extended retention time. However, the olfactory area constitutes only about ~5% of the respiratory region, posing a challenge for effective drug delivery to the olfactory site [130,131]. A considerable amount of the drug tends to settle in the respiratory region post-administration [132], which has a fully functional and active mucociliary clearance system that quickly eliminates drugs, resulting in a brief residence time that hampers the drug’s ability to reach the brain (Figure 3d) [126,133].

In the olfactory system, substances must navigate through the cilia or membranes of olfactory cells, utilizing transmembrane or intercellular transport to cross the olfactory epithelium and access the lamina propria [134]. There are three primary routes for these substances to travel from the lamina propria to the brain (Figure 3I) [128,135,136]. The transcellular route involves drug molecules entering the perineural space of olfactory nerves via mechanisms such as passive diffusion, active transport, facilitated diffusion, or endocytosis, ultimately bypassing the BBB through axonal transport of the olfactory nerves to reach specific brain regions [135]. The paracellular route is characterized by passive transport, where drugs penetrate the olfactory epithelial barrier through junctions between neighboring olfactory epithelial cells, driven by intercellular concentration or electrochemical gradients, allowing access to cerebrospinal fluid or brain tissue [137]. The intracellular route employs olfactory nerve bundles, which are surrounded by olfactory ensheathing cells, as conduits to traverse the cribriform plate in the direction of axonal transport and ultimately reach the brain [136]. Additionally, the respiratory pathway serves as a vital channel for delivering drugs to the brainstem, hindbrain, and spinal cord [138]. Besides transmembrane diffusion, drug molecules can also be taken up by nerve fibers through endocytosis or passive diffusion at the sensory endings of the trigeminal nerve (Figure 3II) [128,139].

### 3.3. Major Factors Influencing Drug Delivery

The efficacy of nasal drug delivery is governed by the intricate interplay among drug physicochemical properties, formulation architecture, and physiological constraints. Molecular weight, lipophilicity, and charge of active compounds critically dictate their permeability across the nasal epithelium and subsequent systemic or central nervous system absorption [140]. Smaller, lipophilic molecules generally exhibit higher transepithelial flux, whereas hydrophilic or macromolecular drugs require advanced carrier systems [141]. Concurrently, the formulation characteristics—including particle size, surface chemistry, and mucoadhesive potential—profoundly influence nasal residence time and mucosal penetration. For instance, Li et al. developed an intranasal conformal patch achieving sustained levodopa release via intimate mucosal contact, underscoring the importance of bioadhesive formulation geometry [142]. Additionally, Zhang et al. demonstrated that thiolated odor-mimetic nanoparticles exploit olfactory receptor interactions to facilitate CNS entry, highlighting the significance of particle surface engineering in nasal delivery design [143].

Beyond formulation design, the nasal physiological environment presents formidable barriers that limit drug bioavailability. The mucociliary clearance apparatus continuously eliminates deposited particles, while the mucus layer and tight junctions of the respiratory and olfactory epithelia create physical obstacles to absorption [144]. Regional heterogeneity within the nasal cavity—particularly the distinction between the vascularized respiratory mucosa that favors systemic absorption and the neural-connected olfactory epithelium that enables direct brain entry—determines the ultimate destination of nasally administered drugs [130]. Yin et al. demonstrated that intranasal administration of a bioengineered nanolamellar system can bypass the blood–brain barrier via sequential mitochondrial targeting, illustrating how anatomical route selection influences therapeutic outcomes [145]. Furthermore, Gao et al. reported that surface-patterned DNA nanocarrier vaccines administered intranasally elicit robust mucosal immunity against respiratory syncytial virus, affirming that overcoming mucosal barriers through rational antigen presentation is essential for nasal vaccination efficacy [146].

To effectively design nasal drug delivery systems, it is essential to thoroughly evaluate the transport routes of drug molecules following nasal administration. This involves the careful integration of strategies for targeting the drug, ensuring biocompatibility and enhancing drug retention properties [147,148]. By optimizing these critical factors in tandem, the delivery system can facilitate the precise, safe, and efficient action of drugs, ultimately enhancing clinical effectiveness while reducing systemic exposure and potential side effects [149,150].

## 4. Critical Considerations of Drug Design

Recent studies in intranasal drug delivery systems have increasingly concentrated on enhancing drug absorption through the precise targeting of the olfactory and trigeminal areas within the nasal cavity. This includes innovations in targeted design [130,151,152], prolonged nasal residence design [153,154,155], biosafety evaluation [26,156,157], and engineering delivery devices for nasal formulations [44,158,159]. Together, these advancements provide solid groundwork for the evaluation and refinement of nasal therapeutics that can be effectively translated into clinical applications [160,161].

### 4.1. Targeted Design

**Figure 4:**
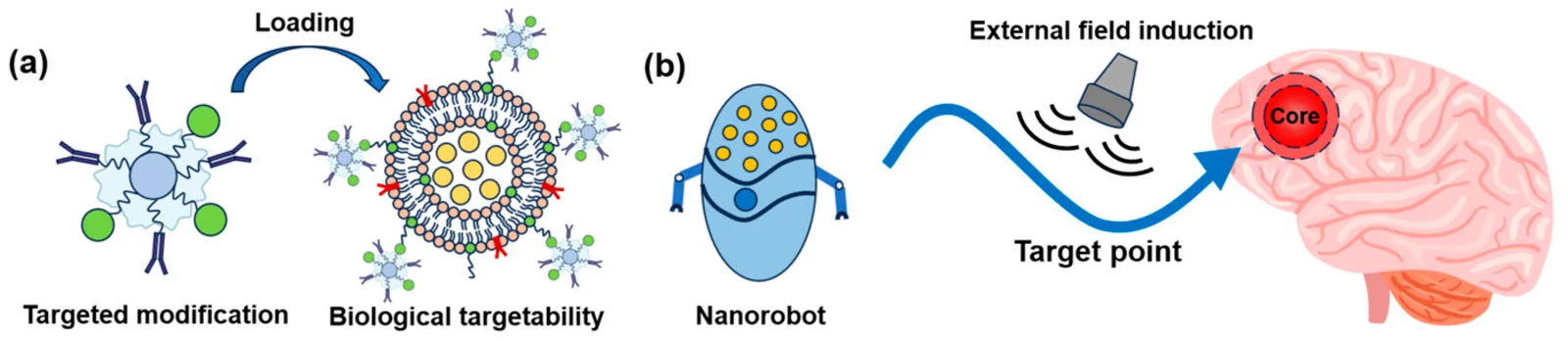
Methods for targeted design of (**a**) targeted ligand modification on biomimetic nanocarriers and (**b**) nanorobot combined with external field-induced technology. Color code: green, targeting ligands; blue, nanocarrier/nanorobot; red, core/target region; yellow, loaded drug.

The design of targeted delivery systems poses significant challenges in the advancement of intranasal therapies [162]. Most therapeutic agents for nose-to-brain delivery are macromolecules, which cannot undergo passive transmembrane transport and rely exclusively on active endocytosis to achieve transepithelial delivery [163]. Additionally, after traversing the biological barriers, the drug must be accurately delivered to cerebral lesion sites, which necessitates sophisticated multi-targeted design and protective modification against degradation during delivery [164,165]. A prominent design strategy, as shown in Figure 4a, involves the modification of biomimetic nanocarriers with targeted ligands [166,167]. This method facilitates the effective and safe transport of therapeutic agents to brain lesion sites while reducing the risk of off-target side effects [168]. In addition, the recently emerging nanorobot combined with external field-induced technology can also enable the specific targeted transport of drug molecules to the brain following intranasal administration (Figure 4b) [37,145,169]. This technology is currently undergoing extensive research and holds promise for future applications in targeted intranasal drug delivery [170].

The creation of targeting ligands and the assessment of their targeting efficacy are essential preliminary studies for the thoughtful development of intranasal therapies, providing a solid theoretical basis for the later design of biomimetic nanocarriers with multiple targeting capabilities [171,172]. In Table 3, recent nasal delivery drugs of targeted design methods are listed. These systems employ diverse strategies to enhance brain delivery. Receptor-mediated targeting (e.g., lactoferrin receptor and rabies virus glycoprotein–nicotinic acetylcholine receptor) facilitates neuronal uptake via receptor-mediated endocytosis and improves therapeutic outcomes in neurodegenerative models [38,173]. Borneol-modified carriers open tight junctions to enhance paracellular permeability and enable deeper brain penetration in glioma and migraine models [174,175]. Stimulus-responsive platforms, including thermosensitive hydrogels and electro-responsive nanodrugs, provide sustained or on-demand release synchronized with pathological activity [176,177]. Biomimetic exosomes offer low immunogenicity, efficient membrane fusion, and selective accumulation in target brain regions [173,178]. PEGylated iron oxide nanoparticles enable dual-mode tracking via magnetic resonance imaging and fluorescence for mechanistic studies [179]. These strategies demonstrate improved brain selectivity and reduced systemic exposure, with DTP reaching 98.55% [176]. The investigations into different target receptors have laid a solid foundation for effective transport via nasal administration. However, most systems remain at the preclinical stage. Challenges include undefined dose–response relationships, long-term nasal mucosa safety concerns, and scalability issues for extracellular vesicle production [176,178].

The strategic development of targeted drug delivery mechanisms allows for the accurate and effective management of disorders affecting the CNS [182]. Nonetheless, many current investigations primarily emphasize the analysis of the functional characteristics of targeting agents or the drugs themselves [183]. To ensure successful transition into pharmaceutical applications, it is crucial to also consider the ease of synthesis and the scalability of production methods for these systems. First, the rational selection of optimal receptors for RMT is a prerequisite for efficient brain delivery. However, the complex trafficking mechanisms and distinct endosomal routing patterns of different RMT receptors make target selection and validation exceptionally difficult [184]. It often results in poor transcytosis efficiency, lysosomal entrapment, or unintended off-target accumulation in peripheral tissues [11]. Second, mounting evidence reveals considerable regional heterogeneity in receptor expression across the BBB endothelium and among various CNS pathologies, introducing significant inter-patient and intra-cranial variability that undermines the universality of any single targeting approach and underscores the need for comprehensive receptor expression profiling prior to therapeutic intervention [185]. Third, receptor saturation represents a critical pharmacological constraint that is frequently overlooked in preclinical studies. High ligand density on nanocarrier surfaces or escalated dosing regimens can overwhelm the limited pool of accessible target receptors, triggering competitive inhibition, diminished brain uptake, and accelerated systemic clearance [10]. Fourth, immunogenicity poses a significant safety concern upon repeated administration. Surface-engineered nanocarriers—particularly PEGylated liposomes and antibody-based shuttles—can elicit anti-drug antibodies, leading to the accelerated blood clearance phenomenon [186]. Additionally, these carriers may trigger complement activation-related pseudoallergy—in severe cases, life-threatening hypersensitivity reactions—thereby compromising both the safety and therapeutic efficacy of the treatment regimen [187]. Furthermore, the clinical manufacturing of targeted nanomedicines demands stringent control over multi-step synthesis, ligand conjugation chemistries, and physicochemical characterization [188]. The current production protocols frequently suffer from batch-to-batch variability, limited scalability under good manufacturing practice (GMP) conditions, and prohibitively high costs [189]. Finally, the regulatory landscape presents significant translational hurdles, as existing drug evaluation frameworks were primarily established for conventional small-molecule pharmaceuticals and biologics rather than multi-component nanomedicines. This will lead to ambiguous classification criteria, inconsistent characterization requirements, and divergent approval pathways among major regulatory agencies [190].

Overcoming these biological, technological, and regulatory challenges requires a coordinated, multidisciplinary effort. Precision target validation is essential to ensure therapeutic specificity, and reproducible manufacturing standards must be established to guarantee batch-to-batch consistency and scalable production. Furthermore, global regulatory frameworks for nanomedicines need to be harmonized to streamline clinical approval pathways. These concerted advances will accelerate the clinical translation of targeted nasal delivery systems for CNS disorders.

### 4.2. Prolonged Nasal Residence Design

The nasal cavity has a restricted area for absorbing medications and its natural mucociliary clearance (MCC) system, which is essential for nasal self-purification [191]. It can quickly remove most drugs delivered intranasally from the absorptive tissue through ciliary action and mucus trapping within approximately 15–20 min [192]. These two physiological limitations collectively result in a substantial reduction in both the absorption efficiency and effective residence time of drugs at the nasal absorption site [193]. To circumvent these barriers, recent investigations have developed diverse sustained-release intranasal formulations broadly categorized into six strategies: mucoadhesive polymers (e.g., chitosan-based systems), mucus-penetrating systems (e.g., lipid–polymer hybrid nanoparticles and lecithin-based micelles), dry powder formulations, in situ gelling systems (e.g., bilosomes and solid lipid nanoparticles in thermo-responsive gel), nanoemulsions, and thermosensitive formulations. Preclinical studies demonstrate that these systems significantly prolong nasal residence time and enhance brain targeting efficiency, with DTE values ranging from 117% to 5451.8% and DTP values up to 98.1% (Table 4). The design of nasal drug delivery systems to enhance the adhesiveness of solvent can greatly extend the time therapeutics remain in the nasal cavity. However, the excessively strong adhesiveness of the drug solvent will restrict the diffusion of drug molecules [194]. This limitation can impede the targeted deposition of active pharmaceutical ingredients (APIs) in the nasal respiratory area, leading to significant premature loss of the drug from the nasal vestibule [195]. A critical appraisal of these formulations reveals distinct advantages and limitations for each approach. Mucoadhesive polymers prolong nasal retention via electrostatic interactions, achieving a mucociliary transit time (MTT) up to 53.3 min and sustained release over 24 h [196,197]. However, excessive adhesion may impede drug diffusion toward the olfactory region. Mucus-penetrating systems (12–98 nm) effectively traverse the mucus barrier [117,198], yet weak adhesion may cause rapid clearance. Dry powders provide superior olfactory deposition and direct nose-to-brain transport [199], but dosing consistency and potential irritation remain concerns. In situ gelling systems achieve extended residence (MTT 22.4 min) and high DTE (up to 5451.8%) [118,200]. Nevertheless, high viscosity may cause nasal obstruction and foreign body sensation. Nanoemulsions exhibit excellent permeability and rapid brain uptake (DTE up to 1317%) [201,202], but instability and potential ciliotoxicity require evaluation. Thermosensitive formulations demonstrate favorable sol–gel transitions and sustained release [203], though complex preparation processes pose scale-up challenges. Clinically, translation of these advanced systems is hindered by inter-individual variability in nasal anatomy, limited patient tolerance to high-viscosity or powder formulations, insufficient long-term safety data, and lack of regulatory guidelines for nanomedicine-based nasal products. Therefore, there is an urgent need for further refinement of methods to enhance the nasal retention of pharmaceutical agents.

**Figure 5:**
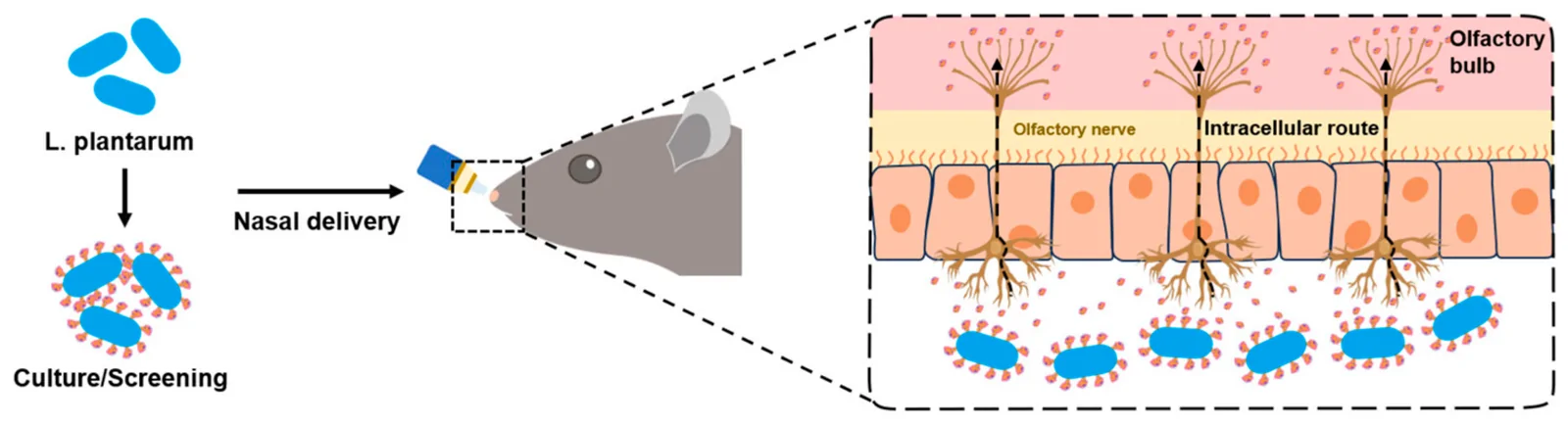
Schematic illustration of the mechanism of engineered bacteria for intranasal drug delivery.

The nasal cavity hosts a remarkably varied community of commensal microorganisms, whose collaborative biological roles help maintain the intricate balance of the intranasal microbial environment [204,205]. While conventional intranasal formulations represent established clinical technologies, the application of synthetic microbial engineering to nasal drug delivery constitutes an emerging proof-of-concept approach in brain-targeted therapies [206]. For instance, Chang and colleagues developed a genetically modified strain of *Lactobacillus plantarum* through targeted alterations [207]. This enhanced probiotic framework facilitates the effective, localized delivery of therapeutic agents to the central nervous system, particularly utilizing the olfactory bulb as a primary site for transit and action (Figure 5). This modified *Lactobacillus plantarum* demonstrates sustained secretory activity in the nasal cavity for more than 14 days, significantly outlasting traditional nasal formulations. This extended retention in the nasal region leads to a notable decrease in the frequency of doses, thereby reducing the likelihood of adverse effects, such as damage to the nasal mucosa from repeated use. However, this strategy remains at an early preclinical stage, and its clinical translation faces substantial biosafety and regulatory hurdles [208]. A comprehensive assessment of the safety profile of this technology is still necessary. In particular, uncontrolled growth of the modified bacterial strain could disrupt the fragile balance of the nasal microbiome, potentially causing harm to the nasal mucosa and adjacent tissues [209]. Additionally, the absence of harmonized regulatory frameworks for engineered live biotherapeutics, coupled with the lack of standardized potency assays under current U.S. Food and Drug Administration (FDA) and European Medicines Agency (EMA) guidance, underscores the gap between preclinical proof-of-concept and clinical implementation [210]. To mitigate this significant concern, it is essential to thoughtfully design a specific genetic regulatory system to keep the bacterial population within a predetermined safe range after administration [207]. Additionally, a reliable and adjustable kill switch should be incorporated into the strain to allow for the termination of its viability and therapeutic function during the treatment period [211].

An alternative strategy to prolong nasal drug residence time is the use of drug-loaded patches for intranasal delivery. This delivery system achieves efficient and sustained release of loaded drugs. Nasal patch-loaded formulations permit an intuitive evaluation of drug release kinetics and pharmacological performance [142]. Owing to its facile operation, this platform can greatly reduce drug loss induced by operational errors. For this innovative drug delivery method to be successfully implemented, several important factors need thorough assessment [212,213]. For biosafety, the biodegradability of the patch and the maintenance of normal respiratory function without causing any sensation of a foreign object should be considered. To increase the drug bioavailability, the kinetics of drug release and the sustained-release characteristics of the therapeutics should be optimized.

### 4.3. Biosafety Evaluation

The biosafety evaluation of nasal drug delivery systems not only provides objective confirmation of formulation safety by assessing various toxicity indicators, but also plays a crucial role in safeguarding clinical translation by reducing harm to the nasal mucosa and ensuring systemic tolerability [214,215]. A thorough assessment of ciliary toxicity, long-term nasal toxicity, mucosal irritation, immunogenicity, repeated dose safety, and histopathological parameters can provide comprehensive evidence for the biosafety profile of nasal formulations, thereby supporting regulatory approval and informed clinical adoption [216]. Establishing robust biosafety profiles is essential for minimizing off-target effects and maximizing therapeutic benefits across diverse patient populations, particularly those with compromised nasal physiology [217].

The biosafety evaluation of nasal drug delivery systems encompasses six critical parameters: ciliary toxicity, long-term nasal toxicity, immunogenicity, repeated dose safety, mucosal irritation, and histopathological evaluation [48,218]. Ciliary toxicity and long-term nasal toxicity assessments ensure the preservation of mucociliary clearance and epithelial integrity during chronic administration [219,220]. Immunogenicity profiling and repeated dose safety studies establish the therapeutic window by evaluating immune responses and cumulative systemic effects [221]. Mucosal irritation assessment and histopathological examination provide direct evidence of local tolerability and tissue compatibility [222,223]. Together, these parameters guide rational formulation optimization and clinical translation. Recent representative examples with key physicochemical and pharmacological parameters are summarized in Table 5.

Biosafety evaluation is a vital criterion for the successful clinical application and verification of nasal drug delivery systems [225]. In the preclinical stage, it is essential to thoroughly assess several key parameters, including the likelihood of nasal mucosal damage from prolonged use of the formulation, the potential for both systemic and localized side effects, ciliary function impairment, immunogenic reactions, and the stability of the delivery system’s physicochemical properties [226]. These comprehensive assessments collectively establish the safety foundation for translating intranasal formulations from bench to bedside, ensuring that only systems with acceptable risk–benefit profiles advance to clinical trials [227]. Furthermore, standardized safety evaluation protocols should be established to facilitate regulatory review and accelerate the clinical translation of promising nasal delivery platforms.

### 4.4. Delivery Condition Optimization Design

The practical use of intranasal drug delivery faces various operational hurdles, lacking the consistency found in intravenous and oral methods [228]. Factors such as differences in nasal mucosal surface area [229] and microbial diversity [230] among humans of varying ages, along with reduced effectiveness in individuals suffering from rhinitis, hinder the standardized use of these drugs. To overcome these technical and translational obstacles, a thorough examination of the ideal parameters for intranasal drug delivery is essential. This includes assessing the angle at which droplets are administered, their ejection speed, the drug’s physicochemical properties at various stages of delivery, and the extent of nasal mucosal coverage [175,231]. A common way is improving the administration angle and spray area for intranasal formulations, resulting in a spray pattern with a narrower plume angle and less spray coverage. This refined approach facilitated the effective delivery of a greater drug quantity to the deep posterior nasal cavity and reduced significant drug loss due to early and excessive deposition in the nasal vestibule, leading to a marked improvement in drug absorption efficiency through the mucosa [175].

Table 6 summarizes the key features of commercially available nasal delivery devices and the multifactorial determinants influencing olfactory deposition in intranasal CNS drug delivery [232,233]. As summarized in Table 6, the investigated devices span conventional pump sprays, bidirectional systems, dry powder inhalers, and novel 3D-printed personalized platforms. The reported olfactory deposition fractions (ODFs) exhibit considerable variability across devices, ranging from 7.11% for bidirectional administration to 23.58% for optimized liquid sprays, and up to 44% for refined unidirectional systems [232]. Critical factors governing olfactory deposition include particle size, spray velocity, nozzle design, spray plume angle, and inspiratory flow rate [233], while device–formulation compatibility with nanocarriers, liposomes, and polymeric nanoparticles represents an additional determinant of delivery efficiency [234]. Notably, patient usability and ergonomic design have emerged as pivotal translational barriers, alongside device–formulation mismatches and individual anatomical variations [235].

Despite substantial advances in intranasal device engineering, the clinical translation of nose-to-brain delivery systems remains largely constrained by insufficient olfactory deposition and inconsistent patient–device coordination [183,235]. Current commercial nasal devices, encompassing conventional pump sprays, bidirectional breath-actuated systems, and dry powder inhalers [232], have demonstrated highly variable ODFs ranging from approximately 7% to 44% in in vitro cast studies [239], highlighting substantial inter-device heterogeneity and the urgent need for standardized evaluation metrics [234]. Clinically, only a limited number of intranasal products have achieved regulatory approval for CNS indications, most notably benzodiazepine rescue therapies for acute seizure clusters [240]. However, the majority of nanoparticle-based carriers, liposomal formulations, and advanced mucoadhesive systems remain in preclinical or early-phase clinical development [183,241]. The principal translational barriers include inter-individual anatomical variability in nasal cavity geometry, suboptimal device–formulation compatibility arising from mismatched spray plume geometry and actuation mechanics, and inadequate patient compliance with proper administration techniques [235,242]. Furthermore, the lack of robust in vitro–in vivo correlation models and the scarcity of large-scale randomized controlled trials have impeded the regulatory endorsement of advanced delivery platforms [183].

Consequently, traditional methods of administering intranasal sprays are limited in their ability to finely tune essential delivery parameters, often resulting in suboptimal drug distribution to the deep posterior nasal cavity [243,244]. Recently, the innovative design of advanced intranasal delivery systems has emerged as an effective approach to overcome this challenge. It focuses on three main areas: enhanced aerosolization, precise drug targeting, and miniaturized device design with simplified operation [43,245]. Beyond enhancing the operational settings of current intranasal delivery systems and creating new delivery devices, effectively optimizing intranasal drug delivery in clinical settings requires tailored device designs that consider the anatomical differences in nasal cavity structures among various patient groups [131,246]. This may involve utilizing scans of individual patients’ nasal anatomy and applying AI-driven algorithms to determine and adjust the ideal parameters for the devices. Such a personalized strategy improves the effectiveness of intranasal drug administration while reducing avoidable drug wastage [247].

## 5. Clinical Evaluation System for Nasal Delivery

### 5.1. Clinical Key Indicators

The use of intranasal delivery has gained attention as a potential method to circumvent the BBB in treating CNS conditions. Although this approach is under significant investigation in various large-scale studies, several key issues need to be thoroughly examined to ensure its effective application in clinical settings moving forward.

**Figure 6:**
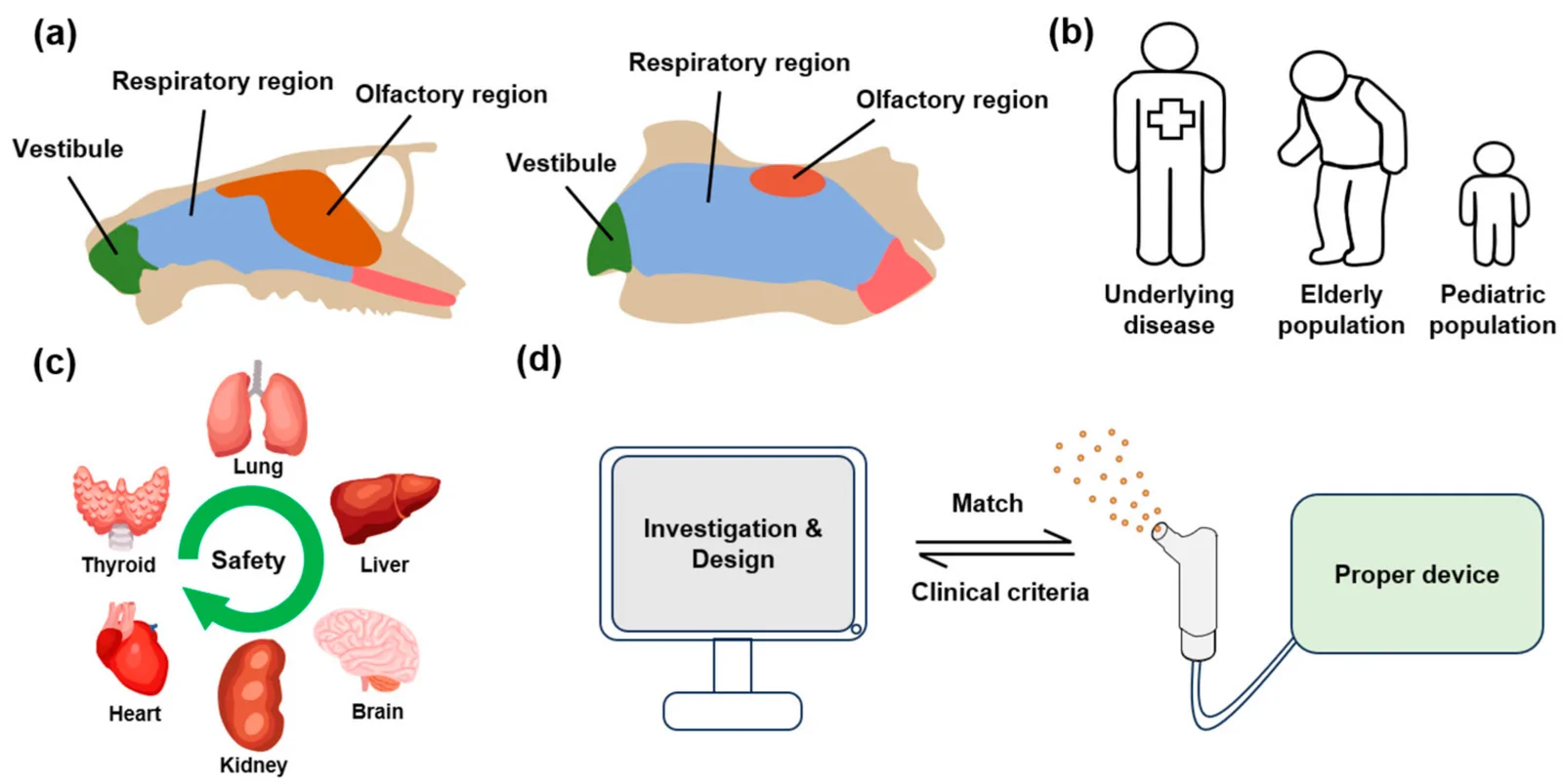
(**a**) Comparative schematic diagram of nasal cavity anatomy in rat and human. Green corresponds to the vestibule, blue represents the respiratory region, light brown indicates the olfactory region, and pink refers to posterior nasal mucosa. (**b**) Patients with underlying diseases and vulnerable populations. (**c**) Safety assessment of nasal formulations across multiple organs. (**d**) Design and manufacture of nasal formulations and associated delivery devices must adhere to rigorous clinical performance metrics.

(1) Precision in targeting: The most distinctive advantage of nasal drug delivery over conventional routes is inherent high targeting specificity compared to oral administration and parenteral injection [248]. Due to the varied nature of lesions associated with neurological disorders, it is essential for nasal drug delivery systems to effectively differentiate and concentrate in the forebrain or hindbrain [130]. Consequently, exploring specific transport routes primarily through the olfactory bulb or trigeminal nerve is crucial for improving both the efficiency of delivery and the precision of targeting in nasal therapies.

(2) Variations in nasal cavity anatomy: The current research and development of new pharmaceuticals predominantly utilize rat models for experimentation. However, there are notable anatomical differences between the nasal cavities of rats and humans [249]. As illustrated in Figure 6a, the olfactory epithelium comprises roughly 50% of the total nasal mucosal area in rats, whereas it constitutes only about 5% in humans. In rats, the respiratory region makes up around 50%, in contrast to approximately 90% in humans [250]. These substantial disparities severely limit the predictive value of rodent-based preclinical studies for human clinical translation [251], as marked differences in olfactory epithelium proportions and respiratory architecture lead to fundamentally divergent mucosal deposition and drug absorption. As a result, large animal models with closer anatomical homology to humans have been increasingly recognized as critical preclinical platforms to improve translational predictability [252]. Furthermore, computational fluid dynamics simulations provide precise visualization of nasal airflow and particulate deposition, enabling the optimization of device design and formulation parameters [253]. In parallel, human nasal replicas enable the reproducible assessment of olfactory-targeting efficiency and regional deposition, serving as complementary tools to bridge the gap between preclinical data and clinical outcomes [254]. Consequently, it is essential to integrate model development with experimental research to establish a connection between rat preclinical studies and human clinical trials, alongside implementing a scoring evaluation framework.

(3) Safety evaluation report: The safety analysis of intranasal treatments should encompass not only healthy individuals, but also detailed assessments for patients with pre-existing conditions, elderly people, and children (Figure 6b) [255]. It is crucial to provide a thorough account of all possible side effects. Beyond evaluating potential harm to the nasal passages and the central nervous system, it is important to investigate the systemic impacts of medications that enter the bloodstream and affect other organs (Figure 6c) [256]. A well-organized and detailed enumeration of a medication’s possible adverse effects is vital for supporting evidence-based and informed treatment decisions across various patient groups.

(4) Scalable manufacturing of nasal products: The manufacture of nasal pharmaceutical products is strictly governed by current GMP requirements, which mandate operation within validated controlled environments—typically Grade A/B cleanrooms for aseptic filling and Grade C/D for preparation and compounding—together with comprehensive environmental monitoring, personnel training, and facility qualification to ensure sterile production and cross-contamination control [257]. As complex nasal formulations such as liposomes, polymeric nanoparticles, and thermosensitive in situ gels transition from laboratory development to commercial scale, they encounter formidable scale-up challenges including non-linear mixing dynamics, heat-transfer variability, and shear-induced degradation, all of which directly compromise the batch-to-batch reproducibility of critical quality attributes including particle size distribution, polydispersity index, zeta potential, and encapsulation efficiency [258,259]. Consequently, rigorous process validation through design-of-experiments and continuous process verification is essential to maintain acceptable intra-batch and inter-batch consistency throughout the product lifecycle. Sterilization constitutes a particularly significant hurdle for these advanced systems because conventional terminal sterilization (e.g., moist heat autoclaving) is incompatible with thermolabile phospholipids, biodegradable polymers, and temperature-sensitive gel matrices; manufacturers must therefore rely on aseptic processing with sterile filtration, gamma irradiation of starting materials, or emerging supercritical CO_2_ technologies, while simultaneously enforcing stringent endotoxin and pyrogen limits validated through limulus amebocyte lysate or rabbit pyrogen tests [260].

(5) Design of devices: Effective nasal drug delivery systems necessitate not just the creation of potent pharmaceutical compounds, but also the incorporation of suitable devices to facilitate optimal drug administration [261]. Throughout the extensive research and development phases for both medications and devices, it is essential to set clinical compatibility criteria to confirm that drug formulations work well with various delivery mechanisms (Figure 6d) [262]. Additionally, pairings of devices and drugs should be categorized based on specific target areas, ensuring that individuals with different neurological conditions can utilize the most effective nasal drug delivery systems available.

(6) Sustaining drug effectiveness: A significant number of nasal spray products consist of potent liposomal or microbe-encapsulated therapies that can successfully penetrate the BBB for extended durations [25,175]. To maintain their therapeutic properties, many of these medications have limited shelf lives or are intended for one-time use [263]. Long-term storage stability is further threatened by physical instabilities such as liposomal fusion, nanoparticle aggregation, Ostwald ripening, and gel phase–transition drift, necessitating validated cold-chain storage (2–8 °C), appropriate antioxidant or cryoprotectant use, and real-time/real-condition stability studies to establish shelf-life specifications [264]. In a clinical setting, it is essential to implement rigorous dosing guidelines and create uniform administration procedures to guarantee that these treatments achieve their optimal therapeutic impact.

(7) Quality Management System: Quality control frameworks for nasal products are equally rigorous, requiring a validated assessment of spray content uniformity, droplet/particle size distribution, spray pattern and plume geometry, delivered-dose uniformity, pH, osmolality, viscosity, and microbial limits, with particular attention to the interference of nanocarriers in standard endotoxin assays and the need for validated analytical methods compliant with International Council for the Harmonization of Technical Requirements for Pharmaceuticals for Human Use (ICH) guidelines [265,266]. Finally, regulatory approval demands adherence to evolving international standards, including Quality by Design implementation aligned with ICH principles, comprehensive device–formulation compatibility studies, and harmonized post-approval change management protocols to ensure reproducible clinical performance and patient safety [267].

### 5.2. Advances in Clinical Research on Intranasal Drug Delivery

Clinical applications of intranasal administration have witnessed notable breakthroughs and advances in recent years. Table 7 summarizes the current landscape of representative intranasal delivery platforms, comparing their key advantages, limitations, scale-up feasibility, and clinical maturity. Liposomes offer high biocompatibility and dual hydrophilic/lipophilic encapsulation but suffer from low mucosal stability and rapid mucociliary clearance [38,268]. Polymeric nanoparticles provide tunable size, charge, and sustained release, yet they face batch variability and a translational gap beyond rodent models [269,270]. Nanoemulsions enhance hydrophobic drug solubility and mucosal penetration but remain exclusively preclinical with undefined regulatory pathways for RNA [271,272]. Hydrogels enable in situ gelation to prolong nasal residence, and poloxamer-based products have already reached the market, whereas novel composites are still preclinical [273,274]. Exosomes demonstrate innate biocompatibility, low immunogenicity, and natural BBB crossing ability, yet large-scale GMP manufacturing and standardized purification remain bottlenecks [275,276]. Biomimetic carriers achieve immune evasion and prolonged circulation, but industrial-scale coating and complex isolation limit their progress [277]. Dry powders offer cold-chain-free storage and scalable spray-drying technology, with engineerable particle size for olfactory targeting, thus possessing the highest scale-up feasibility among the platforms reviewed [229,278].

Most advanced intranasal delivery systems remain in the preclinical stage, with only hydrogels and exosomes progressing into early clinical trials (Phase I/II) [274,276]. No platform has yet reached late-stage clinical development or regulatory approval for CNS delivery. Scale-up feasibility varies considerably: dry powders and conventional hydrogels are relatively mature, whereas exosomes, nanoemulsions, and biomimetic carriers face substantial manufacturing and standardization challenges. These findings highlight the persistent gap between preclinical innovation and clinical translation in intranasal drug delivery, underscoring the urgent need for standardized nasal epithelium models, GMP-compliant processes, and robust regulatory frameworks to advance nose-to-brain therapeutics [279].

In terms of the clinical feasibility of formulations, Table 8 summarizes recent advances in intranasal CNS formulations, ranging from two U.S. FDA-approved benzodiazepine nasal sprays for seizure clusters (Nayzilam^®^ [Upsher-Smith, Minneapolis, MN, USA] and Valtoco^®^ [Neurelis, San Diego, CA, USA]) to Phase II/III randomized controlled trials of intranasal insulin for Alzheimer’s disease and type 2 diabetes-related cognitive impairment [280,281,282]. Various preclinical platforms, including thermosensitive hydrogels [176], exosome nanocarriers [178], microneedles [283], and RNA cell-penetrating peptide nanocomplexes [284], have shown efficacy in animal models. Yet only NEO100 (perillyl alcohol) has reached Phase II for brain tumors [285]. Beyond neurodegenerative diseases, intranasal delivery has achieved regulatory milestones in broader CNS disorders. Esketamine nasal spray (Spravato^®^ [Janssen Pharmaceuticals, New Brunswick, NJ, USA]) received FDA approval in 2019 for treatment-resistant depression following Phase III trials, demonstrating rapid antidepressant onset within 24 h [286]. Zavegepant nasal spray, a selective calcitonin gene-related peptide receptor antagonist, received FDA approval in 2023 following positive Phase III evaluation for acute migraine, achieving pain freedom in 24% of patients versus 15% with placebo at 2 h post-dose [287]. These successes underscore the clinical translational potential of nasal administration. Nevertheless, widespread adoption for CNS indications remains constrained by interspecies anatomical differences, mucociliary clearance, variable epithelial permeability, and the necessity for optimized permeation enhancers to ensure reproducible nose-to-brain transport in humans [288].

Despite these advances, critical translational challenges persist. Nose-to-brain delivery efficiency remains disappointingly low (<1% for most formulations), while rodent–human nasal anatomical differences (olfactory epithelium ~50% vs. ~5%) and undefined regulatory pathways for nanoscale formulations impede clinical translation [291]. To accelerate progress, integrated strategies are warranted: the standardization of delivery devices, incorporation of non-human primate models and 3D human nasal epithelium for preclinical validation, biomarker-driven patient stratification (e.g., apolipoprotein genotype), and early regulatory engagement to clarify nanocarrier safety expectations [279]. The convergence of formulation science, device engineering, and precision medicine will be essential to bridge the gap between bench and bedside for intranasal CNS therapeutics.

### 5.3. Quantitative Clinical Evaluation Framework for Nasal Drug Delivery

To address the qualitative limitations of the current evaluation framework and to align with international regulatory standards, a five-domain standardized scoring system for nasal drug delivery (SDSS-NDD, 0–100 scale) is proposed. This scoring system integrates quantitative pharmacokinetic endpoints, measurable efficacy indicators, safety metrics, and translational benchmarks consistent with FDA, EMA, and ICH guidelines.

(1) Targeting Precision (20 points): This domain evaluates the anatomical targeting specificity of nasal formulations, distinguishing between olfactory-bulb-directed (forebrain) and trigeminal-nerve-directed (hindbrain) delivery. Quantitative indicators include (i) olfactory deposition ratio (ODR), measured using gamma scintigraphy or computational fluid dynamics modeling; (ii) regional brain concentration index, defined as the ratio of drug concentration in the target brain region (olfactory bulb, striatum, hippocampus) versus plasma at time to peak concentration (T_max_); and (iii) DTP, calculated as the proportion of brain drug uptake attributable to non-systemic (nasal-to-brain direct) pathways. Based on FDA and EMA bioequivalence guidance for locally acting nasal products, an ODR ≥ 30% and DTP% ≥ 15% are considered predictive of meaningful nose-to-brain targeting [292,293].

(2) Pharmacokinetic Performance (25 points): To evaluate this domain, according to ICH and FDA bioequivalence guidance [292,294], the following endpoints are established as primary quantitative metrics: (i) C_max_ (peak plasma/brain concentration) and T_max_ (time to peak); (ii) area under the curve from zero to last measurable concentration (AUC_0_–t) and area under the curve from zero to infinity (AUC_0_–∞) (systemic and central exposure); (iii) terminal half-life (t1/2); (iv) drug targeting efficiency (DTE); (v) drug transport percentage (DTP); and (vi) cerebrospinal fluid (CSF) concentration ratio (CSF/plasma). For nasal CNS delivery, a DTE > 150% and DTP > 20% indicate favorable direct brain targeting relative to intravenous administration. CSF/plasma ratios > 0.5 at T_max_ support olfactory/trigeminal transport dominance over systemic redistribution [294].

(3) Safety Evaluation (25 points): Safety assessment follows the tiered framework recommended by ICH and FDA preclinical guidance for nasal drug products [292,295]. Local safety metrics include (i) ciliary beat frequency preservation (≥80% of baseline indicates acceptable safety); (ii) MCC time (<20 min ideal; >30 min indicates ciliotoxic risk); (iii) nasal mucosa histopathology score (adapted from the quantitative scoring system for nasal mucosa scale: 0 = normal, 4 = severe damage); (iv) olfactory function (University of Pennsylvania Smell Identification Test score ≥ 30/40 = normal; <25 indicates clinically significant olfactory dysfunction); and (v) local tolerability index integrating erythema, edema, and epithelial integrity. Systemic safety metrics include the incidence of treatment-emergent adverse events, serious adverse events, and drug-related discontinuation rates. Formulation pH should be maintained at 4.5–6.5 and osmolarity at 200–600 mOsm/L to preserve mucosal integrity (EMA guideline) [296].

(4) Formulation and Device Performance (15 points): This domain evaluates physicochemical and device parameters critical for clinical translation, as specified in FDA chemistry, manufacturing, and controls guidance and the EMA 2024 inhalation/nasal quality guideline [292,293]. Key indicators include (i) delivered dose uniformity (coefficient of variation <10% across devices); (ii) droplet/particle size distribution (optimal range 10–50 μm for olfactory targeting, <10 μm risks pulmonary deposition); (iii) spray pattern and plume geometry (ellipticity ratio 1.0–1.5); (iv) emitted dose (≥85% of label claim); (v) device robustness (drop test, re-priming consistency), and (vi) formulation stability (particle size, zeta potential, and drug loading within ± 10% of initial values over 24 months).

(5) Translational Readiness (15 points): Translational benchmarks bridge preclinical and clinical development, addressing anatomical scaling and model validation. Quantitative indicators include (i) species bridging index, comparing olfactory epithelium surface area ratios (rat 50% vs. human 3%), requiring allometric scaling correction (body surface area^0.67^); (ii) preclinical-to-clinical pharmacokinetics correlation (R^2^ ≥ 0.70 for interspecies area under the curve (AUC) prediction); (iii) biomarker alignment (CSF or plasma biomarker changes consistent between species); (iv) GMP readiness score; and (v) clinical trial readiness (ethics approval and patient stratification criteria). A translational readiness score ≥10/15 indicates that preclinical data are sufficiently robust to support an investigational new drug (IND) application.

The total score (0–100) is interpreted as follows: ≥85: Excellent translational readiness—suitable for accelerated IND submission and Phase I initiation; 70–84: Good—standard IND pathway with defined risk mitigation; 55–69: Moderate—requires additional preclinical validation before clinical entry; 40–54: Limited—significant gaps in PK, safety, or device performance; major remediation needed; <40: Insufficient—not recommended for clinical translation without fundamental reformulation or redesign. This scoring framework provides a reproducible, evidence-based tool for comparing nasal drug delivery candidates and prioritizing development candidates with the highest probability of clinical success. The domain weights were assigned according to each domain’s relative contribution to clinical translation, as informed by regulatory guidelines and evaluation literature. PK performance and safety received the highest weights (25 points each), as PK endpoints (C_max_, AUC, drug-targeting efficiency) constitute primary registrational criteria in FDA/EMA guidance [292,293], while safety data serve as the principal gatekeepers for first-in-human approval [294]. Targeting precision (20 points) reflects the central rationale of nose-to-brain delivery [23,24]; formulation/device performance and translational readiness (15 points each) are enabling domains governed by FDA CMC guidance and GMP requirements [292]. The five-tier rubric was benchmarked against the GRADE framework [297]. These expert-informed estimates warrant future validation via formal consensus methods (e.g., Delphi studies) [298].

The clinical translation of nasal drug delivery remains promising, yet faces substantial fundamental and translational challenges that must be addressed in the coming decade. As mentioned above, biological barriers (e.g., mucociliary clearance, enzymatic degradation, and interindividual variability in nasal anatomy and physiology) pose significant obstacles to the consistency and reproducibility of intranasal drug delivery [116]. Furthermore, the significant discrepancy between animal models and human nasal anatomy may lead to the overestimation of formulation efficacy in preclinical studies, necessitating careful validation before clinical implementation [57]. Despite these challenges, the ongoing development of advanced nanoparticle formulations and engineered delivery systems, coupled with comprehensive safety assessments and the integration of artificial intelligence for personalized protocol design, is expected to progressively enhance the clinical viability of nasal drug delivery within the next 10 to 15 years [207,299]. However, the rapid clinical transformation of nasal drug delivery will require the further elucidation of anatomical transport mechanisms, standardization of administration devices, and rigorous demonstration of efficacy in well-designed clinical trials [300,301].

## 6. Conclusions

While numerous recent reviews have cataloged advances in nose-to-brain delivery technologies, the present work distinguishes itself by systematically connecting basic research breakthroughs with clinical implementation through a structured evaluation framework. Nasal drug administration offers a non-invasive approach with significant potential for treating CNS disorders by circumventing the BBB. This route eliminates hepatic first-pass metabolism, minimizes systemic toxicity, and enhances brain drug availability. This review integrates the current understanding of olfactory and trigeminal transport mechanisms with design strategies spanning targeted ligands, prolonged nasal residence, biocompatible materials, and optimized delivery devices. Its central contribution is a clinical evaluation framework that assesses targeting precision, pharmacokinetic performance, multi-organ safety, device–drug compatibility, and anatomical translation. By applying this framework, we identify critical bottlenecks—including the need for region-specific targeting strategies, insufficient clinical data for vulnerable populations, and the lack of standardized device compatibility criteria—that must be resolved to advance clinical translation. Looking forward, converging advances in pharmaceutics, materials science, molecular biology, and artificial intelligence will enable the development of increasingly personalized nasal delivery systems. The proposed evaluation framework provides a practical roadmap to align laboratory innovations with clinical requirements, thereby accelerating the transition of intranasal drug delivery from experimental models to routine patient care for CNS disorders.

## Figures and Tables

**Table 2:** Brain targeting assessment parameters of various drugs following intranasal administration.

Drug	Route/Formulation	DTE	DTI	DTP	Brain/Plasma C_max_ Ratio	Relative Brain Bioavailability	Ref.
Teriflunomide (TEF)	IN Lipid Carriers	357.55%	3.58	72.03%	1.78	135.72 ± 10.56%	[119]
Clozapine (CLZ)	IN Micelles	117%	6.03	83.42%	2.96	170.59%	[117]
Zolpidem (ZP)	IN Lipid Carriers	368.69 ± 10.30%	144.58 ± 10.22	99.17 ± 0.04%	9.85	487.78 ± 97.66%	[120]
Metoclopramide (MTC)	IN Nano-Vesicular	198.55%	4.06	75.26%	1.35	295.02%	[123]
Artemether (ARM)	IN Lipid Carrier	502.7%	5.02	0.04%	2.33	132.95 ± 10.56%	[124]
Clonazepam	IN Polymeric Micelles	242.3%	144.25	99.3%	5.03	812.96 ± 154.6%	[121]
Zolmitriptan	IN Bilosome	5451.8%	/	98.1%	4.15	1173.64%	[118]
Mirtazapine (MZP)	IN Lipid Nanocapsules	332.2%	10.73	90.68%	3.29	509.28%	[125]
Quetiapine Fumarate (QF)	IN Lipid Carrier	455.76 ± 10.25%	NR	85.26 ± 6.45%	0.70	356.87 ± 19.38%	[122]

Note: DTE, Drug Targeting Efficiency; DTI, Drug Targeting Index; DTP, Drug Transport Percentage; C_max_, Maximum Observed Concentration; IN, Intranasal; NR, not reported.

**Table 3:** Nasal delivery drugs of targeted design with different methods.

Targeting Strategy	Rationale	Transport Mechanisms	Improvement vs. Non-Targeted	Limitations	Brain Accumulation	Therapeutic Outcome	Ref.
Lf-LNPs (α-Mangostin + si*BACE1*)	LfR Overexpression; RMT-Mediated BBB Crossing	RMT (LfR); Olfactory/Trigeminal Nerves	Enhanced Aβ Clearance; *BACE1* Silencing	Preclinical; Mucus Barrier; Liver Tropism (IV)	Reduced Aβ Plaques/Neuroinflammation	Cognitive Recovery; Aβ Reduction; Oxidative Stress Attenuation	[38]
Borneol/R8dGR-PLGA NPs (Curcumin + Cisplatin)	Borneol: TJ Opening; R8dGR: Integrin αvβ3 Targeting	TJ Disruption (Borneol); RMT (R8dGR-αvβ3)	Higher Glioma Targeting; Deeper Penetration; Prolonged Survival	Preclinical; Borneol Dose Optimization	Enhanced Brainstem Accumulation; Improved Permeation	Hypoxia Attenuation; Angiogenesis Inhibition; Survival Prolongation	[174]
Borneol-LNP Nasal Spray (BLNP-NS)	Borneol: Nasal TJ Opening; Viscosity-Optimized for olfactory deposition	Olfactory Deposition; Mucosal Absorption; TJ Modulation	Accelerated Onset; Superior Efficacy vs. Conventional Spray	Preclinical; Viscosity Optimization Required for Individual Nasal Anatomy	Higher ODF; ODF-Intracerebral Correlation	Rapid Migraine Relief; Hyperalgesia Reversal; Microglial Polarization Regulation	[175]
RVG-Engineered EVs (*FGF20*-Loaded)	RVG-nAChR Neuronal Targeting; *FGF20* Neuroprotection	RME (RVG-nAChR); i.v. → BBB Crossing	Superior Brain Delivery; Enhanced Neuronal Uptake	Labeling Limitations; Dose–Response Undefined	Enhanced Accumulation; Efficient Ischemic Region Delivery	Neuroplasticity; Functional Recovery (Motor/Cognitive)	[173]
PEGylated Fe_3_O_4_ NPs (Fluorescent-Labeled)	PEGylation: Aggregation Prevention; Fe_3_O_4_: MRI-Guided Tracking	Perineural Transport (Olfactory/Trigeminal); CSF Circulation	Improved Distribution; MRI/Fluorescence Dual Tracking	Mechanistic Study; no Therapeutic Cargo	Multi-Region MRI/Fluorescence/ICP-MS Quantification	Mechanistic Insights for Intranasal Nanomedicine Optimization	[179]
Thermosensitive Hydrogel (Intranasal BP-MB)	MB: Tau Aggregation Inhibitor; BP: ROS Scavenger	Mucoadhesive Retention; Transcytosis across Nasal Epithelium	Enhanced Bioavailability; Sustained Release; Improved Cognition vs. free MB	Preclinical Stage; Scalability of BP NSs Synthesis; Long-Term Nasal Mucosa Safety	Higher Brain AUC; Reduced Peripheral Exposure (DTP 98.55%)	Cognitive Amelioration; Mitochondrial Restoration; Neuroinflammation Inhibition	[176]
Thermosensitive Nanogel–Hydrogel Nasal Drops (DGND)	SA: Sirt1 Activator; Nanogel: MMP-Responsive Release	Olfactory Transport; Transcellular Permeation	Superior Brain-Targeting; Sustained Release	Nasal Cavity Volume Limit; Preclinical Stage; Long-Term Safety	Enhanced Accumulation; Prolonged Hippocampal Retention	Cognitive Dysfunction Attenuation; Neuroinflammatory Marker Reduction	[177]
RVG29-MSC Exosomes	RVG29-nAChR Targeting; Membrane Fusion; Low Immunogenicity	RMT (RVG29-nAChR)	Enhanced SN Accumulation; Improved Neuronal Uptake; Superior Motor Recovery	EV Production Scalability; Batch Variability	SN-Selective Accumulation; TH+ Neuronal Colocalization; Minimal off-Target	PD Motor Alleviation; TH Normalization; Neuroinflammation Suppression	[178]
Electro-Responsive Nanodrugs (Intranasal)	P-Peptide Mucosal Penetration; T-Peptide Neuronal Targeting	Electric-Responsive Release; T-Peptide/GT1B-Mediated Neuronal Uptake	Rapid Onset; Seizure-Matched Release; Enhanced Seizure Control	Preclinical Stage	Olfactory Pathway; Seizure-Synchronized Release	Seizure Suppression; Locomotor Recovery; Neuroprotection	[180]
Thermosensitive Chitosan Hydrogel (Cell Therapy)	Chitosan: Mucoadhesion + TJ Modulation; Cell/Cargo Protection	Mucoadhesive Retention; Chitosan-Mediated TJ Opening	Enhanced Cell Viability (>95%); Improved Delivery vs. Liquid	Chitosan MW-Dependent Kinetics; Scalability of Functionalization	Prolonged Retention (>72 h); Enhanced Accumulation in Olfactory Bulb/Cortex	Cargo Brain Delivery; Prolonged Cell Viability; Mucopenetration Enhancement	[181]

Note: AUC, area under the curve; Aβ, amyloid-beta; *BACE1*, *beta-secretase 1*; BLNP-NS, borneol-modified lipid nanoparticle nasal spray; BP, black phosphorus; CSF, cerebrospinal fluid; DGND, drop to gate nasal drops; DTP, drug transport percentage; EVs, extracellular vesicles; *FGF20*, *fibroblast growth factor 20*; GT1B, ganglioside GT1b; ICP-MS, inductively coupled plasma mass spectrometry; IV, intravenous; Lf, lactoferrin; LfR, lactoferrin receptor; LNPs, lipid nanoparticles; MB, methylene blue; MMP, matrix metalloproteinase; MRI, magnetic resonance imaging; MSC, mesenchymal stem cell; MW, molecular weight; nAChR, nicotinic acetylcholine receptor; NPs, nanoparticles; NS, nasal spray; NSs, nanospheres; ODF, olfactory drug fraction; PD, Parkinson’s disease; PEG, polyethylene glycol; PLGA, poly(lactic-co-glycolic acid); R8dGR, R8-dGR peptide; RME, receptor-mediated endocytosis; RMT, receptor-mediated transport; ROS, reactive oxygen species; RVG, rabies virus glycoprotein; RVG29, rabies virus glycoprotein 29; SA, sirtuin activator; si*BACE1*, small interfering RNA against *BACE1*; Sirt1, sirtuin 1; SN, substantia nigra; TH, tyrosine hydroxylase; TJ, tight junction; αvβ3, alpha-v beta-3 integrin.

**Table 4:** Comparison of key parameters for sustained-release intranasal drug formulations.

Formulation Type	Particle Size	Drug Loading	Brain Accumulation Efficiency	Application/Disease	Residence Time	Translational Status	Ref.
Nanoemulsion O/W NE	153.2 nm	85.72%	DTE 1317%, DTP 92.40%	Cerebral Ischemia	1.47 h (Brain Mean Residence Time)	Preclinical (rats)	[201]
Mucus-Penetrating System LPH NPs	97.56 ± 4.55 nm	8.57 ± 0.54%	DTE 394.94%, DTP 74.68%	Schizophrenia	Sustained Release 24 h (in vitro)	Preclinical (rats)	[198]
Mucus-Penetrating System LbPM	12.23 ± 4.76 nm	6.47 ± 0.15%	DTP 83.42%	Schizophrenia	Sustained Release 24 h (in vitro)	Preclinical (mice)	[117]
Thermosensitive Formulation CS NPs in Poloxamer Gel	130.6 ± 8.38 nm	61 ± 0.89%	DTI 1.27	Cerebrovascular Disorders	Sustained Release 24 h (in vitro)	Preclinical (rats)	[203]
In Situ Gel Bilosomes in Mucoadhesive Gel	399.80 ± 4.95 nm	70.34 ± 0.10% (Drug Entrapment Efficiency)	DTE 5451.8%, DTP 98.1%	Migraine	22.36 ± 0.41 min (Mucociliary Transit Time)	Preclinical (rats)	[118]
In Situ Gel SLN in Thermo-Responsive ISG	358.53 ± 18.24 nm	15%	DTE 137.54%, DTP 27.29%	Parkinson’s Disease	21.7 min (Nasal Residence)	Preclinical (rats)	[200]
Mucoadhesive Polymer Chitosan NPs in Situ Gel	180 ± 1.5 nm	11 ± 0.3%	DTE 1177.3%, DTP 91.5%	Epilepsy	53.3 ± 11.5 min (Mucociliary Transit Time)	Preclinical (rats)	[196]
Nanoemulsion Chitosan-Coated NE	68 ± 1 nm	85.5 ± 0.3% (Drug Entrapment Efficiency)	DTE 510.90%, DTP 80.39%	Neuroblastoma	Sustained release 24 h (in vitro)	Preclinical (rats)	[202]
Dry Powder Spray-Dried Microparticles	106–500 µm	34.6 ± 2.3%	DTE 456%, DTP 78%	Alzheimer’s Disease	/	Preclinical (rats)	[199]
Mucoadhesive Polymer Chitosan NPs	167 ± 6.5 nm	32.25 ± 1.63%	DTE 508.59%, DTP 80.34%	Depression	Sustained Release 24 h (in vitro)	Preclinical (rats)	[197]

Note: DTE, Drug Targeting Efficiency; DTP, Drug Transport Percentage; DTI, Drug Targeting Index; NE, Nanoemulsion; O/W, Oil-in-Water; LPH, Lipid–Polymer Hybrid; NPs, Nanoparticles; LbPM, Lecithin-based Polymeric Micelles; CS, Chitosan; SLN, Solid Lipid Nanoparticles; ISG, In Situ Gel.

**Table 5:** Biosafety evaluation strategies for nasal drug delivery.

Safety Evaluation Parameter	Sample	Particle Size	Drug Loading	Brain Enrichment Efficiency	Therapeutic Efficacy	Translational Stage	Ref.
Ciliary Toxicity	Hyaluronan-Enveloped Nanomicelles	105.7 nm	siRNA Binding Efficiency >98.4%	Significantly Higher Brain Accumulation vs. Uncoated	Median Survival 17–18 Days; Significant Tumor Growth Inhibition	Preclinical in vivo (Mice for Efficacy; Rats for Safety)	[219]
Long-Term Nasal Toxicity	CircRNA-Loaded Lipid Nanoparticles	140–170 nm	EE > 90%	IN Significantly Higher than IV in Peri-infarct Region	Improved Sensorimotor Function, Cognition, Synaptic Plasticity	Preclinical in vivo (Rats)	[217]
Histopathological Evaluation	Bioengineered Nanolamellar System	~224 nm	pH 6.7 Release: FTY720 ~84%; PolyMet ~66%	4.27-Fold vs. Bare; 77% Nasal Epithelium Permeability	In vitro: Apoptosis 74.56% → 7.47%; in vivo: M2/M1 Ratio 0.15 → 1.21	Preclinical in vitro/in vivo (Rats)	[145]
Ciliary Toxicity	Lyotropic Liquid Crystalline Nasal Spray	/	5 mg/kg (LDA IN)	26-Fold Higher Brain Bioavailability vs. Oral	Significant Improvement in Behavioral and Cognitive Disorders	Preclinical (Rats and Toads)	[220]
Repeated Dose Safety	Single-Molecule Multifunctional Scaffold	~1–2 nm	/	Significantly Higher Brain Fluorescence vs. Free Drug	Aβ↓74%; Cognition DI↑3.8×; TUNEL+↓87%; TNF-α↓44%	Preclinical in vivo (Mice)	[224]
Mucosal Irritation	Curcumin-Loaded Liposomal Nanoparticles	~70 nm	EE: 76.2%	1.6-Fold Higher Brain Fluorescence vs. IV	Neuronal Apoptosis 33.8% → 10%; Aβ Clearance; M1 → M2 Polarization	Preclinical in vitro/in vivo (Mice)	[222]
Mucosal Irritation	Odorranalectin-Modified Lipid Nanoparticles	104.75 nm	siRNA EE 93.5%; QU DL 7.44%	1.74-Fold Brain Fluorescence vs. Unmodified; Trans-Barrier + 78.4%	Reduced Aβ Plaques; Improved Cognition and Spatial Memory	Preclinical in vitro/in vivo (Mice)	[223]
Immunogenicity	Biomineralized Virus-like Particles	~84.4 nm	/	Significantly Higher Accumulation in Glioma Tissue	Significant Antiglioma Efficacy; Robust, Durable Tumor Suppression	Preclinical in vitro/in vivo (Mice)	[221]

Note: EE, Encapsulation Efficiency; IN, Intranasal; IV, Intravenous; LDA, Levodopa; QU, Quercetin; DL, Drug Loading; Aβ, Amyloid-β; DI, Discrimination Index; TUNEL+, TdT-mediated dUTP Nick-End Labeling Positive; TNF-α, Tumor Necrosis Factor-alpha; M1, Classically Activated Macrophages; M2, Alternatively Activated Macrophages; FTY720, Fingolimod; PolyMet, Polymetformin; siRNA, Small Interfering RNA; CircRNA, Circular RNA. Footnote: Arrows in the table represent the change trend of indicators. ↑ means increase, ↓ means decrease, and → indicates the transformation of numerical values before and after treatment.

**Table 6:** Key features of commercial nasal devices and factors affecting olfactory deposition in intranasal CNS drug delivery.

Commercially Available Nasal Delivery Devices	Deposition Technologies	Patient Usability	Device–Formulation Compatibility	Factors Influencing Olfactory Deposition	Ref.
Pump Sprays; Bidirectional Devices; Clinical Translational Potential	Regional Deposition Patterns; Spray Plume Geometry; Actuation Mechanics	Ergonomic Design; Minimal Training; Improved Compliance	Nanocarriers; Permeability Enhancers; Formulation Stability During Actuation	Particle Size; Spray Velocity; Nozzle Design	[236]
Standard Nasal Spray; Borneol-Modified Lipid Nanoparticles	ODF 23.58% (F5); ODF–brain delivery correlation (R^2^ = 0.7755)	Liquid Spray; Self-Administration; Repeated Dosing Suitable for Chronic CNS Diseases	Dispersion Medium Viscosity; Nasal Deposition; Nose to Brain Efficiency	Droplet Size; Dispersion Viscosity; Spray Plume angle	[175]
Novel Nasal Devices; Translational Pipeline for Alzheimer’s, Parkinson’s, Stroke and Brain Tumors	Olfactory Deposition Mechanisms; 3D-Printed Nasal Cast Evaluation	Patient Compliance and Ease of Use as Critical Translational Barriers	Liposomes; Polymeric Nanoparticles; Compatibility with Various Device Platforms	Anatomical; Physiological; Formulation-Related Factors	[228]
Nanotechnology-Integrated Nasal Devices; Advanced Carrier Systems for Neurological Diseases	Nasal Deposition Patterns of Nanoformulations; Olfactory Mucosal Deposition Enhancement	Non-Invasive; Improved Acceptability vs. Parenteral Routes	Optimal Particle Size (5–20 μm); Surface Charge; Mucoadhesive Properties	Formulation Parameters (Size, Charge, Density); Device Parameters (Spray Geometry, Velocity)	[237]
Uni-Directional vs. Bi-Directional Devices; 3D-Printed Nasal Casts	Uni-Directional: 22.33% ODF (vs. 7.11% Bi-Directional); Optimized up to 44%	Dry Powder; Ease of Handling; Storage Stability vs. Liquid	Powder Flow Properties; Device Airflow Resistance; Deposition Outcomes	Instillation Angle; Left vs. Right Nostril Deposition Asymmetry; Inspiratory Flow Rate	[232]
Dry Powder Nasal Devices; in vitro Deposition Platforms for Nose to Brain	Higher Inspiratory Flow Reduces Surface Coverage but Enhances Olfactory Dose	Patient Coordination for Breath-Actuated Devices; Flow Rate-Dependent Performance	Excipient Effects (Lactose, Albumin); Particle Engineering; Olfactory and Turbinate Deposition	Dry Powder Aerosol Properties; Inspiratory Flow; Nasal Airway Dilation (2.2–4-Fold Increase)	[234]
Spray Pump Modifications; Regional Nasal Drug Delivery; Olfactory Targeting	Regional Deposition Patterns; Olfactory vs. Respiratory Region Distribution	Spray Plume Orientation; Patient-Independent Performance; Minimal Coordination Requirements for Regional Deposition	Spray Plume Geometry; Particle Size; Actuation Mechanics; Device–Formulation Interaction	Spray Plume Angle; Droplet Size Distribution; Actuation Force	[233]
Nose to Brain Therapeutic Devices; Clinically Tested and Commercially Available Platforms	Olfactory Deposition Insufficiency as Major Cause of Clinical Trial Failures	Poor Compliance and Improper Technique Contribute to Suboptimal Outcomes	Integrated Device–Formulation Co-Design; Mismatches between Formulation and Device Output	Inadequate Device–Formulation Matching; Individual Anatomical Variation	[238]

Note: ODF, olfactory deposition fraction; R^2^, coefficient of determination; F5, fifth group.

**Table 7:** Comparative overview of representative intranasal drug delivery platforms.

Delivery Platform	Key Advantages	Key Limitations	Scale-Up Feasibility	Clinical Maturity	Ref.
Liposomes	High Biocompatibility; Dual Hydrophilic/Lipophilic Encapsulation; Surface Engineering for Nose-to-Brain Targeting	Low Mucosal Stability; Rapid Mucociliary Clearance; Exclusively Preclinical	Moderate. Lipid Extrusion is Scalable; Targeted Variants Require Validated Ligand Conjugation	Preclinical. No Liposome-based Intranasal Product in Phase III	[38,268]
Polymeric Nanoparticles	Biodegradable FDA-Approved Polymers; Tunable Size/Charge; Controllable Sustained Release; Amenable to Functionalization	Batch Variability; Translational Gap beyond Rodents; Lack of Standardized Nasal Epithelium Models	Moderate. Raw Materials are Commercial; Large-Scale Multi-Functional Production Remains Challenging	Preclinical. No Polymeric NP Intranasal Formulation in Late-Stage Trials	[269,270]
Nanoemulsions	Enhanced Hydrophobic Drug Solubility; Nanoscale Droplets Facilitate Mucosal Penetration; Thermoresponsive Variants Sustain Release	Exclusively Preclinical; Temperature Control Needed; Limited Stability; Undefined Regulatory Pathways for RNA	Low–Moderate. Homogenization Mature, Thermoresponsive/RNA Variants Add Complexity	Preclinical. No Nanoemulsion-Based Intranasal CNS Product in Trials	[271,272]
Hydrogels	Responsive to Nasal Microenvironment; In Situ Gelation Prolongs Residence	Preclinical for Novel Compositions; pH Variability Affects Release	Moderate. Poloxamer Gels Scalable; Responsive Systems Need Optimization	Early Clinical. Poloxamer Products Marketed; Novel Composites Preclinical	[273,274]
Exosomes	Innate Biocompatibility and Low Immunogenicity; Natural BBB-Crossing Ability; Tissue-Specific Targeting Tropism; Versatile Cargo Loading	Unstandardized Large-Scale Isolation; High Batch Heterogeneity; Variable Loading Efficiency; Sourcing Constraints	Low–Moderate. GMP Manufacturing and Standardized Purification Remain Bottlenecks	Preclinical to Early Clinical (Phase I/II). No Exosome-Based Intranasal Product Approved	[275,276]
Biomimetic Carriers	Immune Evasion, Natural Homing, Prolonged Circulation; Multimodal Integration	Batch Variation; Complex Isolation/Coating; Immunogenicity Risk; GMP not Established	Low. Industrial-Scale Barrier; High-Throughput Coating not Achieved	Preclinical. No CMBNs in Clinical Trials	[277]
Dry Powders	Cold-Chain-Free; Spray-Drying Mature; Particle Size/Spray Angle Engineerable for Olfactory Targeting	Sex/ethnicity-Dependent Anatomical Variability; Thermal Stress Challenges Protein Stability; Particle Bounce Requires Coating	High. Spray Drying Scalable; Device Standardization Supports Commercialization	Preclinical Proof-of-Concept	[229,278]

Note: FDA, Food and Drug Administration; RNA, Ribonucleic Acid; GMP, Good Manufacturing Practice; CMBNs, Cell Membrane Camouflaged Biomimetic Nanomedicines.

**Table 8:** Recent advances in intranasal formulations for CNS applications.

Formulation	Disease	Clinical Feasibility	Translational Challenges	Stage	Ref.
Midazolam Nasal Spray (Nayzilam^®^)	Seizure Clusters	Phase 3 RCT; 53.7% vs. 34.3% Placebo (*p* = 0.011); 72.4% Regained Baseline within 24 h	Age ≥ 12 Limitation; Respiratory Depression Risk; ≤5 uses/month; Long-Term Tolerability Data Limited	FDA-Approved (Phase III)	[289]
Diazepam Nasal Spray (Valtoco^®^)	Seizure Clusters	Phase 3 Open-Label; 12.6% Required Second Dose; 71.8% Retention over 17.4 Months; no Treatment-Related SAEs	Bioavailability vs. Rectal Diazepam; Nasal Irritation; Long-Term Mucosal Effects; RCT Confirmation Needed	FDA-Approved (Phase III)	[290]
Regular Insulin Nasal Spray (40 IU/day)	Alzheimer’s Disease/Mild Cognitive Impairment	Phase 2/3 RCT (*n* = 289); Primary Endpoint ns; Device 1 Cohort (*n* = 49) Cognitive Benefits; no CSF Differences	Device Consistency; Dose–Response Undefined; *APOE* ε4 Stratification; Long-Term Hippocampal Effects Unclear	Phase II/III RCT	[280]
Insulin Nasal Spray (24 Weeks)	T2DM/Cognitive Impairment	Phase II RCT (*n* = 244); Gait/CBF↑ in T2DM, Cognition↑ in Controls; Heterogeneous; Dropout 17%	Adherence; Benefit Differential; Long-Term Outcomes	Phase II RCT	[281]
Insulin Nasal Spray (WMH Analysis)	Alzheimer’s Disease/Mild Cognitive Impairment	Phase 2 Secondary; 12-Month INI Slowed WMH Progression; WMH Linked to Cognitive/CSF Decline; Insulin Protects white Matter	Limited Sample; WMH Surrogate Validation; MRI/CSF Integration Needed	Phase II RCT	[282]
Esketamine Nasal Spray (Spravato^®^)	Treatment-Resistant Depression	Phase 3 RCT; FDA Approved 2019; MADRS ↓ −4.0 vs. Placebo (*p* = 0.010); Rapid Onset within 24 h; 51% Relapse Risk Reduction at 16 Weeks	REMS Requirement; Sedation/Dissociation Risk; Transient BP Elevation; Abuse Potential; Monitoring Burden; Long-Term Efficacy >1 Year Unclear	FDA-Approved (Phase III)	[286]
Zavegepant Nasal Spray	Acute Migraine	Phase 3 RCT (*n* = 1405); 24% vs. 15% Pain Freedom at 2 h (*p* < 0.001); 40% vs. 31% MBS Freedom at 2 h (*p* = 0.001); Onset 15 min; Sustained Relief to 48 h; No Hepatotoxicity	Dysgeusia (21%); Nasal Discomfort (4%); Single-Attack Design Limits Long-Term Tolerability; Consistency Across Multiple Attacks Unconfirmed; Active Comparator Trials Needed	FDA-Approved (Phase III)	[287]
Dissolvable Microneedle Patch (HA-GT + Lf-st-MOF/Hup A)	Alzheimer’s Disease	Rapid Dissolution; AChE/Aβ/ERK-CREB-BDNF Modulation; Memory Improvement; no Ciliary Damage	Needle Length; Self-Administration; Chronic Inflammation; Drug-Loading Limit	Preclinical (Rats)	[283]
PEG-PGA RNA–CPP Nanocomplexes	Neurodegenerative Diseases	miR-132 N-to-B delivery to Hippocampus; Modulates mRNA Targets; Non-Viral RNA Delivery	Scalable Production; CPP Immunogenicity; Rodent-Only; Repeated-Dose Tolerance	Preclinical (Mice)	[284]
BP-MB Thermosensitive Hydrogel	Alzheimer’s Disease	In Situ Gelation; 24 h Nasal Retention; BBB Bypass; Tau/Mitochondrial/Neuroinflammation Modulation; Cognition Improvement	Long-Term Biocompatibility; Scalability; Dose Optimization; Manufacturing Consistency	Preclinical (Mice)	[176]

Note: AChE, acetylcholinesterase; *APOE*, *apolipoprotein E*; BDNF, brain-derived neurotrophic factor; BP, blood pressure; BP-MB, black phosphorus–methylene blue; CBF, cerebral blood flow; CPP, cell-penetrating peptide; CSF, cerebrospinal fluid; ERK, extracellular signal-regulated kinase; HA, hyaluronic acid; HA-GT, hyaluronic acid and tannic acid-crosslinked gelatin; Hup A, huperzine A; INI, intranasal insulin; IU, international unit; Lf-st-MOF, lactoferrin-functionalized stigmasterol-reinforced γ-cyclodextrin-based metal–organic framework; MADRS, Montgomery–Åsberg Depression Rating Scale; MBS, most bothersome symptom; MOF, metal–organic framework; ns, not significant; PEG, polyethylene glycol; PGA, polyglutamic acid; RCT, randomized controlled trial; REMS, Risk Evaluation and Mitigation Strategy; SAEs, serious adverse events; T2DM, type 2 diabetes mellitus; WMH, white matter hyperintensities. Footnote: Arrows in the table represent the change trend of indicators. ↑ means increase, ↓ means decrease.

## Data Availability

No new data were generated or analyzed in this study. All data supporting the findings are available from published public literature.

## Abbreviations

The following abbreviations are used in this manuscript (in the main text):

WHO	World Health Organization
CNS	Central nervous system
BBB	Blood–brain barrier
BTB	Blood–tumor barrier
MCC	Mucociliary clearance
APIs	Active pharmaceutical ingredients
DTE	Drug targeting efficiency
DTI	Drug Targeting Index
DTP	Drug transport percentage
C_max_	Maximum observed concentration
AUC	Area under the curve
AUC_0_–t	Area under the curve from zero to last measurable concentration
AUC_0_–∞	Area under the curve from zero to infinity
CSF	Cerebrospinal fluid
EMA	European Medicines Agency
FDA	Food and Drug Administration
GMP	Good Manufacturing Practice
IND	Investigational new drug
ICH	International Council for Harmonisation of Technical Requirements for Pharmaceuticals for Human Use
MTT	Mucociliary transit time
ODF	Olfactory deposition fraction
ODR	Olfactory deposition ratio
PK	Pharmacokinetics
RMT	Receptor-mediated transcytosis
RNA	Ribonucleic acid
TfR	Transferrin receptor
T_max_	Time to peak concentration
WNT	Wingless-related integration site
